# Selective constraints in cold‐region wild boars may defuse the effects of small effective population size on molecular evolution of mitogenomes

**DOI:** 10.1002/ece3.4221

**Published:** 2018-07-21

**Authors:** Jianhai Chen, Pan Ni, Thuy Nhien Tran Thi, Evgeniy Varisovich Kamaldinov, Valeriy Lavrentyevich Petukhov, Jianlin Han, Xiangdong Liu, Nikica Šprem, Shuhong Zhao

**Affiliations:** ^1^ Key Lab of Agricultural Animal Genetics and Breeding Ministry of Education College of Animal Science and Veterinary Medicine Huazhong Agricultural University Wuhan China; ^2^ The Cooperative Innovation Center for Sustainable Pig Production Huazhong Agricultural University Wuhan China; ^3^ Department of Ecology and Evolution University of Chicago Chicago Illinois; ^4^ National Institute of Animal Sciences Hanoi Vietnam; ^5^ Federal State Budgetary Educational Institution of Higher Education Novosibirsk State Agrarian University Novosibirsk Russia; ^6^ International Livestock Research Institute (ILRI) Nairobi Kenya; ^7^ CAAS‐ILRI Joint Laboratory on Livestock and Forage Genetic Resources Institute of Animal Science Chinese Academy of Agricultural Sciences (CAAS) Beijing China; ^8^ Department of Fisheries, Beekeeping, Game Management and Special Zoology Faculty of Agriculture University of Zagreb Zagreb Croatia

**Keywords:** Ka/Ks ratio, mitogenome, purifying selection, selective constraint, *Sus scrofa*

## Abstract

Spatial range expansion during population colonization is characterized by demographic events that may have significant effects on the efficiency of natural selection. Population genetics suggests that genetic drift brought by small effective population size (*N*_e_) may undermine the efficiency of selection, leading to a faster accumulation of nonsynonymous mutations. However, it is still unknown whether this effect might be balanced or even reversed by strong selective constraints. Here, we used wild boars and local domestic pigs from tropical (Vietnam) and subarctic region (Siberia) as animal model to evaluate the effects of functional constraints and genetic drift on shaping molecular evolution. The likelihood‐ratio test revealed that Siberian clade evolved significantly different from Vietnamese clades. Different datasets consistently showed that Siberian wild boars had lower Ka/Ks ratios than Vietnamese samples. The potential role of positive selection for branches with higher Ka/Ks was evaluated using branch‐site model comparison. No signal of positive selection was found for the higher Ka/Ks in Vietnamese clades, suggesting the interclade difference was mainly due to the reduction in Ka/Ks for Siberian samples. This conclusion was further confirmed by the result from a larger sample size, among which wild boars from northern Asia (subarctic and nearby region) had lower Ka/Ks than those from southern Asia (temperate and tropical region). The lower Ka/Ks might be due to either stronger functional constraints, which prevent nonsynonymous mutations from accumulating in subarctic wild boars, or larger *N*_e_ in Siberian wild boars, which can boost the efficacy of purifying selection to remove functional mutations. The latter possibility was further ruled out by the Bayesian skyline plot analysis, which revealed that historical *N*_e_ of Siberian wild boars was smaller than that of Vietnamese wild boars. Altogether, these results suggest stronger functional constraints acting on mitogenomes of subarctic wild boars, which may provide new insights into their local adaptation of cold resistance.

## 1 INTRODUCTION

Evaluating the relative contributions of selection and genetic drift in shaping genome evolution is one of the central issues in population and evolutionary biology. Although previous studies have suggested the complementary effects of selective constraints and genetic drift on the molecular evolution of mtDNA (Mitterboeck & Adamowicz, 2013; Shen, Shi, Sun, & Zhang, 2009), there is still a lack of compelling cases in which opposite effects of genetic drift and selective constraints are observed. It is feasible to differentiate their effects by comparing populations with different effective population sizes (*N*_e_) and natural selection intensity. Population genetics has predicted that the efficacy of purifying selection on mutations is sensitive to *N*_e_ (Kimura, 1985). In particular, population with a smaller *N*_e_ would be less effective in removing slightly deleterious mutations in comparison with population with a larger *N*_e_, resulting in a faster accumulation of nonsynonymous mutations (Ohta, 1973). To evaluate the relative effects of genetic drift and selective constraints, it is also important to choose a proper genetic system. In this study, mitogenomes were used, as they have only 1/4 *N*_e_ that of nuclear genes (Hard & Clark, 1989). The relatively lower *N*_e_ might facilitate the detection of selection pressure based on the inference of population genetics. In addition, in terms of gene functions, mitochondria are well identified as the power station of cells for thermogenesis and thermoregulation upon which the basic physiological processes are possible (Block, 1994; Rowland, Bal, & Periasamy, 2015; Skulachev, 1999). Owing to these strong functional constraints in mitochondria, the evolution of mtDNA is maintained mainly by purifying selection to remove the accumulation of deleterious nonsynonymous mutations (Sun, Shen, Irwin, & Zhang, 2011). Hence, it is appropriate to use mtDNA as a model to examine the relationship between the effects of genetic drift and functional constraints.

In general, the tempo of molecular evolution may be shaped by selective constraints and *N*_e_ (Kimura, 1985). As most nonsynonymous mutations are (or at least) slightly deleterious (Kryukov, Pennacchio, & Sunyaev, 2007; Mezmouk & Ross‐Ibarra, 2014), the metric of Ka/Ks, representing the proportion of nonsynonymous mutations relative to synonymous mutations, has commonly been used to indicate the efficacy of selection (Hughes, 2013; Wang et al., 2011). In particular, for mitogenomes, which are mainly subject to purifying selection (Ka/Ks < 1) against deleterious mutations (Stewart, Freyer, Elson, & Larsson, 2008), higher Ka/Ks ratios in a population indicate a faster accumulation of slightly deleterious mutations and less efficiency of selection (Björnerfeldt, Webster, & Vilà, 2006; Wang et al., 2011). Previous studies have found ample cases in which the relaxation of selective constraints and/or reduction in *N*_e_ result in the faster accumulation of slightly deleterious mutations. For example, the birds and insects with weaker locomotive abilities have higher Ka/Ks in their mitogenomes than those with stronger locomotive abilities (Mitterboeck & Adamowicz, 2013; Shen et al., 2009). Domestic dog and yak also evolve under higher Ka/Ks in mitochondrial genes compared with their wild counterparts (Björnerfeldt et al., 2006; Wang et al., 2011). These observations have been interpreted by either the relaxation of functional constraints on mitochondrial genes or the reduction in *N*_e_ (Björnerfeldt et al., 2006; Mitterboeck & Adamowicz, 2013; Shen et al., 2009; Sun et al., 2011; Wang et al., 2011). However, hitherto very few evidence has been reported to show which factor, for example, selective constraints or *N*_e_, would have larger effects on the tempo of molecular evolution, if the effects of selection and *N*_e_ on molecular evolution are opposite.

**Figure 1:**
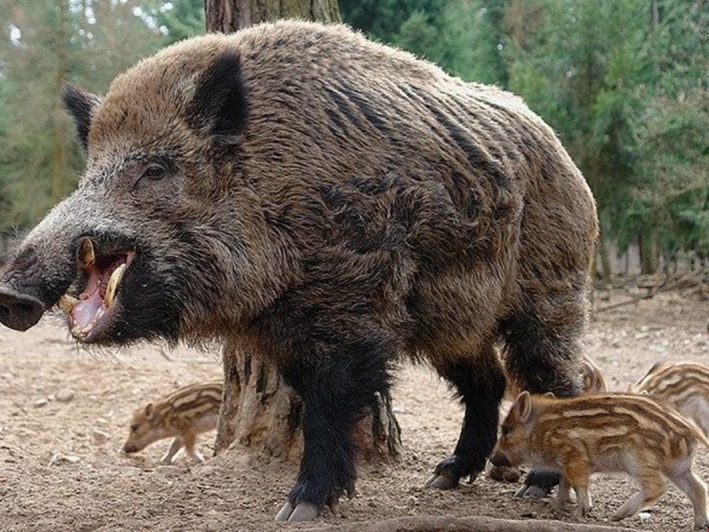
Illustration of wild boars in Novosibirskaya Oblast (*Sus scrofa*). Illustration by Dmitriy Valeryevich Kropachev

In this study, we chose wild boar (and free‐range domestic pigs; *Sus scrofa*) as a model (Figure 1), due to not only their complex natural and artificial selection history, but also their demographic changes along the process of selections. As one of the most successful animals in spreading from Southeast Asia to Eurasia, wild boars are well adapted to various different ecological conditions and environments, including torrid tropical areas, humid forests, arid semideserts, and freezing subarctic winters (Rothschild & Ruvinsky, 2011). Unlike tropical areas, cold regions may impose more stringent evolutionary constraints on mitochondria due to its important role in thermal physiology, leading to the expectation of a slower accumulation of slightly deleterious mutations in mitochondrial genes. However, considering the effect of “Out‐of‐Southeast Asia” dispersal or limited gene flow due to geographic isolation, *N*_e_(*s*) of wild boars in cold regions are expected to be smaller. This rationale may lead to an opposite inference that purifying selection in cold regions might be less effective to remove deleterious mutations than that in tropical areas due to genetic drift. Therefore, wild boar may serve as an intriguing proxy to deepen our understanding about the relationship between the effects of genetic drift and selective constraints in influencing the evolution of protein‐coding genes.

Here, the mitogenomes of wild boars and free‐range local domestic pigs from a tropical region (Son La province, Vietnam) and three subarctic regions in Siberia (Tyva, Zabaykalsky Krai, and Novosibirsk) were sequenced and compared. The major objectives of this study include the following: (a) based on phylogenetic structure of both Vietnamese and Siberian wild boars (and local pigs) to evaluate the potential differences in tempos of accumulation of slightly deleterious mutation; and (b) to evaluate the causes of these differences in the context of selective constraints and *N*_e_.

## 2 MATERIALS AND METHODS

### 2.1 DNA samples and sequencing of mitogenomes

**Figure 2:**
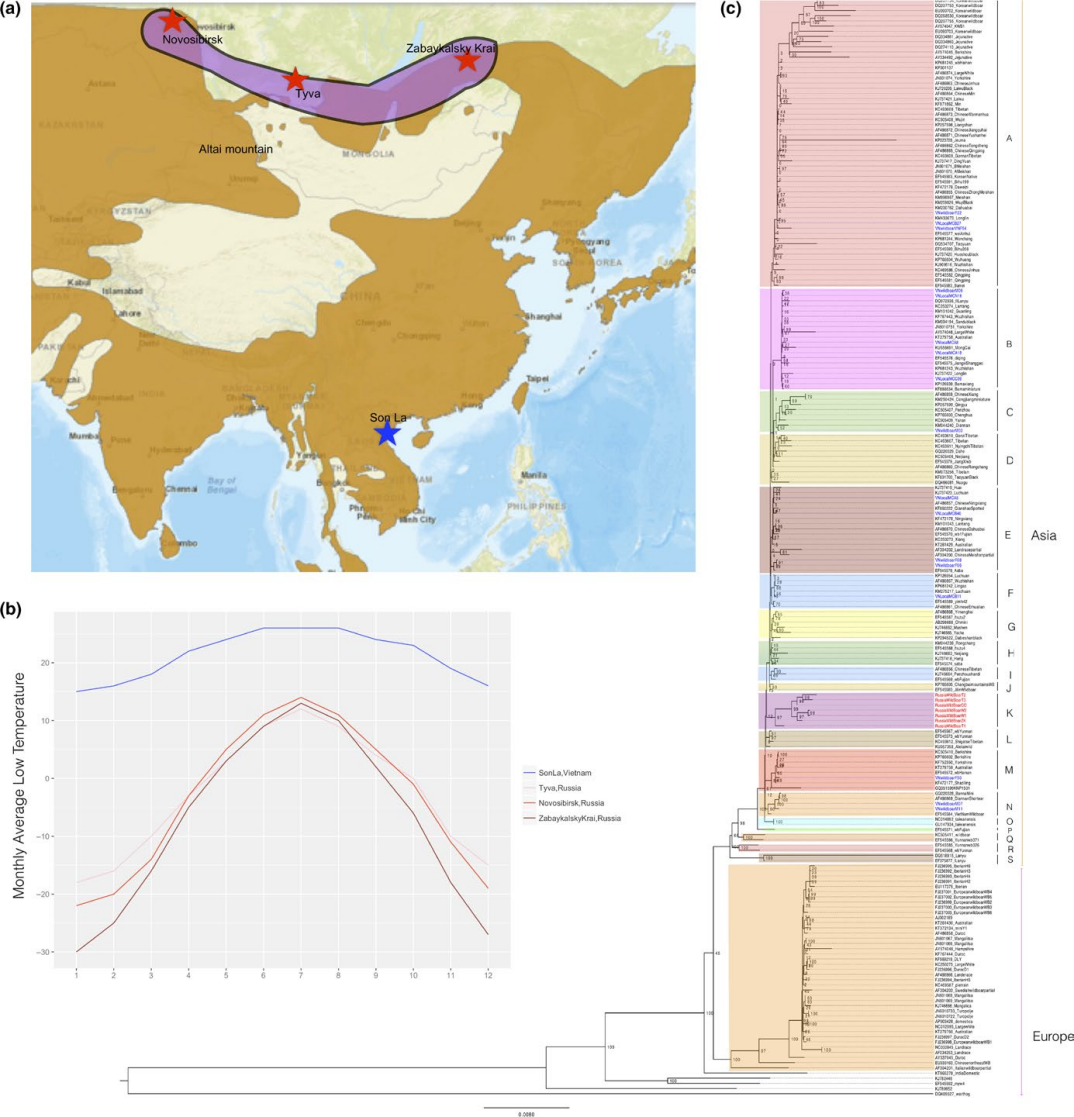
(a) Sample localities. The brown regions are current distributions of wild boars from IUCN (http://maps.iucnredlist.org/map.html?id=41775). (b) Monthly average low temperature (°C) of three sampling locations in Siberia and one location in Vietnam. Data were extracted from http://www.worldweatheronline.com/. (c) Maximum‐likelihood tree of domestic pigs and wild boars from Eurasia based on complete mtDNA sequences. An African warthog (GenBank: DQ409327) was used as the outgroup. The red taxa are Siberian wild boars (subclade K), and blue lineages are Vietnamese wild boars and domestic pigs

In this study, we sequenced the mitogenomes of wild boars and free‐range domestic pigs representing geographic samples spanning the northern‐ and southern‐most ranges of nonintrogressed wild boars and local pigs, with a latitude difference of over 30°. Nine wild boars and eight free‐range pigs from the tropical Vietnam (Son La province, ~20°N) and seven wild boars from subarctic Siberia (three from Tyva, ~51°N, two from Zabaykalsky Krai, ~53°N, and two from Novosibirsk, ~55°N) were surveyed (Figures 1 and 2a). It is worth noting that, to date, there is no evidence to show Siberia as one of the domestication centers for pigs, so it seems impractical for us to incorporate any traditional local breeds from this region. Thus, for Siberian samples, only wild boars can be used. In addition, to alleviate the potential problem of limited diversity caused by smaller sample size of Siberian wild boars, we sampled wild boars from three regions with long distances between each other (>1,400 km between Tyva and Zabaykalsky Krai; >1,100 km between Novosibirsk and Tyva). The sampling locations for Vietnam and Siberia are very different in the monthly average low temperatures (Figure 2b). For all samples, we extracted total DNA from frozen ear tissues using TIANamp Genomic DNA Kit (DP304). Complete genome sequences were amplified using the previously reported primers (Wu et al., 2007). PCR products were purified on spin columns and then sequenced using the ABI 3730 DNA Sequencer (Applied Biosystems, Foster City, CA, USA). Sequencing data were edited using the DNASTAR multiple program package (DNASTAR Inc., USA). All 24 complete mitogenomes have been submitted to GenBank with accession ID from KX982629 to KX982652. All experimental procedures were approved by the Institutional Animal Care and Use Committee of Huazhong Agricultural University, Wuhan, China (permit HZAUSW2015‐0003). In addition, we de novo assembled 24 mitogenomes from publicly available next‐generation sequencing (NGS) data (>25) using the ORGanelle ASeMbler software (http://pythonhosted.org/ORG.asm/). We removed the adapters and low‐quality reads using Trimmomatic (Bolger, Lohse, & Usadel, 2014) and conducted two rounds of de novo assembling. First, the default parameters were set to generate raw sequences. Second, by fine‐tuning our parameters, including the read length, seed sequence, and the input read numbers, we make sure that all sequences were circle. Then, the assembling graphs were built and unfolded to generate complete mtDNA sequences. These sequences were aligned with the reference sequence (NCBI Reference Sequence: NC_000845.1) and manually corrected the orientation of fragments. The annotation was conducted using BLAST comparison (McGinnis & Madden, 2004).

### 2.2 Phylogenetic and network reconstruction

**Figure 3:**
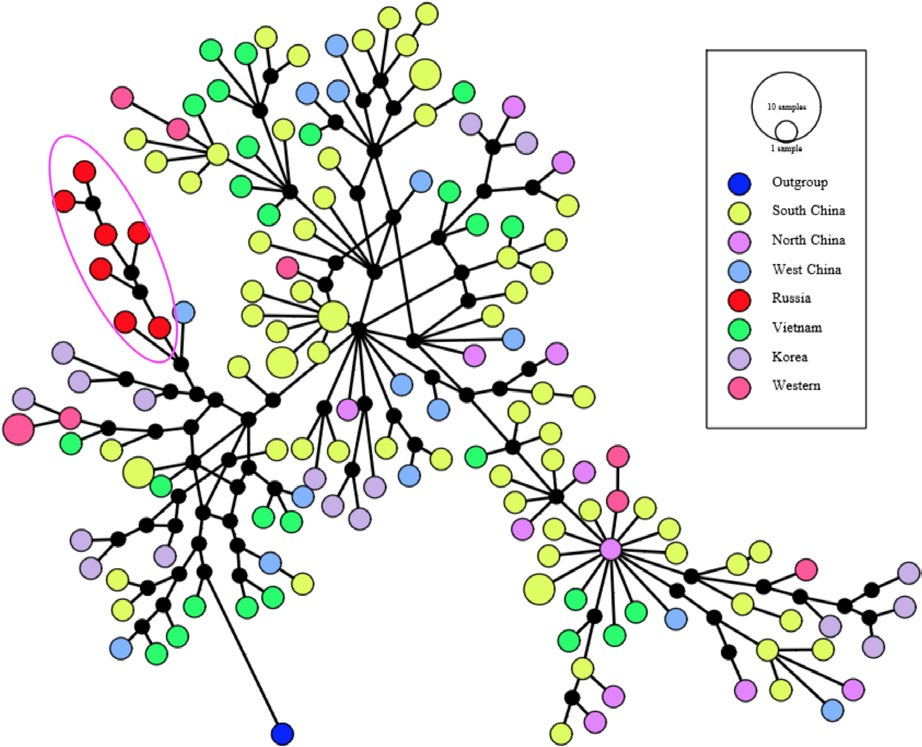
Parsimony networks constructed for complete mitogenomic sequences using the median‐joining method (Bandelt et al., 1999) as implemented in PopART v.1 (Leigh & Bryant, 2015). The Siberian subclade is highlighted within the big circle

The haplotypes of all complete mitogenomes were determined using DnaSP v5 (Librado & Rozas, 2009). To determine the phylogenetic positions of Siberian wild boars and Vietnamese wild boars and free‐range local domestic pigs, we conducted neighbor‐joining (NJ), maximum likelihood (ML), and Bayesian inference (BI) for all Eurasian complete mitogenomes (187 Sanger sequencing data and 24 NGS data, details are shown in Supporting information Additional file 2). An African warthog (*Phacochoerus africanus*, GenBank: DQ409327) was used as an outgroup. The sequences were aligned with MAFFT (Katoh, Misawa, Kuma, & Miyata, 2002) and checked with MEGA6 (Tamura, Stecher, Peterson, Filipski, & Kumar, 2013). Duplicated sequences were detected and removed with RAxML 8.0.20 (Stamatakis, 2014). NJ tree was inferred using MEGA6 with Tamura–Nei method and a GAMMA distribution (shape = 0.9730), as recommended by jModeltest v2.1.7 (Darriba, Taboada, Doallo, & Posada, 2012; Tamura et al., 2013; Supporting information Additional file 1: Figure S1). ML inference was conducted using RAxML 8.0.20 with the “GTRGMMAI” model, which was the second best model inferred from jModeltest v2.1.7 (shape = 0.9730; Pinvar = 0.4790) (Darriba et al., 2012) (Figure 2c). Supportive values were estimated using 1,000 bootstrap replicates. BI was conducted with MrBayes v3.2 (Supporting information Figure S2; Ronquist et al., 2012). Following the suggestion in the manual of MrBayes v3.2, we used a truly Bayesian approach by sampling across substitution models (Ronquist et al., 2012). Parsimony haplotype networks were constructed for complete mitogenomic sequences using the median‐joining method (Bandelt, Forster, & Röhl, 1999) as implemented in PopART v.1 (Leigh & Bryant, 2015) (Figure 3).

### 2.3 Ka/Ks calculation

**Figure 4:**
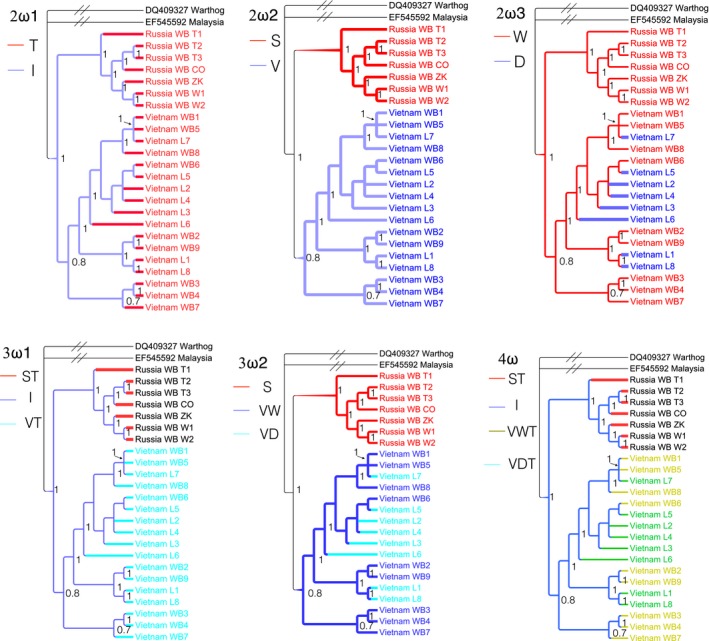
Models used in Ka/Ks calculation. In addition to “1ω” model and “Fω” model, other six models are also used in LRT of branch models. Branches with different colors are different in Ka/Ks ratios. The tree used for LRT was generated from the Bayesian inference. The number around nodes are supportive values. “T,” “I,” “S,” “V,” “W,” and “D” represent “Terminal branches,” “Internal branches,” “Siberian branches,” “Vietnamese branches,” “Wild boars,” and “Domestic pigs,” respectively. A warthog and a Malaysian wild boar were used as outgroups

**Figure 5:**
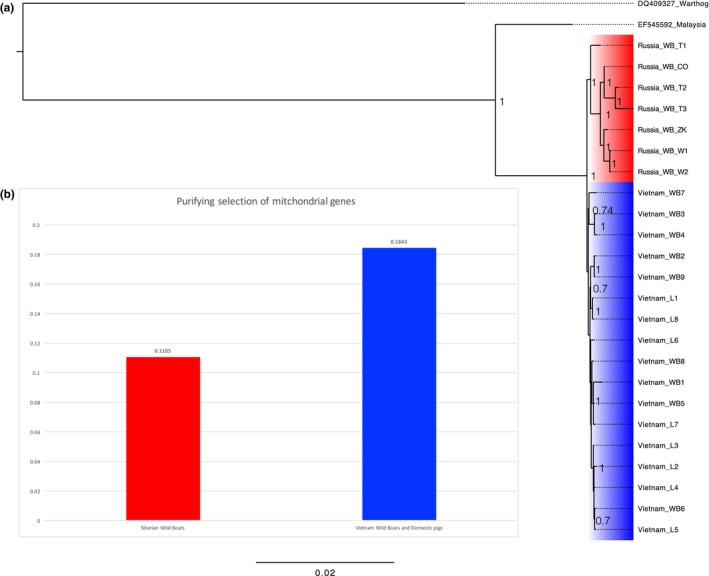
(a) The phylogenetic tree of Siberian wild boars and Vietnamese wild boars and domestic pigs inferred with Mrbayes 3.2. (b) The intensity of purifying selection of mitochondrial genes. The red and blue represent Siberian wild boars and Vietnamese wild boars and domestic pigs (Mongcai pigs), respectively

In 13 mitochondrial genes, as *ND6* has a different codon usage biases (Hasegawa, Cao, & Yang, 1998) and was thus excluded, the other 12 protein‐coding genes and their combined dataset were analyzed independently. The ML method implemented in the program CODEML of PAML was used to evaluate Ka/Ks with F3X4 codon frequencies (Yang, 2007). Two datasets were assembled to evaluate the differences between Ka/Ks ratios of Vietnamese and Siberian samples: (a) Vietnamese and Siberian wild boars (VS_w_, 16 samples); (b) Vietnamese wild boars, Siberian wild boars, and Vietnamese free‐range local pigs (VS_wf_, 24 samples). For VS_w_ data, as the sample sizes of Vietnamese and Siberian wild boars were comparable, we calculated two types of Ka/Ks: pairwise Ka/Ks and phylogeny‐based Ka/Ks. The pairwise Ka/Ks ratios were averaged separately for Vietnamese and Siberian wild boars. As a more powerful method than pairwise sequence comparison (Angelis, dos Reis, & Yang, 2014), the phylogeny‐based method was processed by model comparison (Supporting information Tables S2–S14). Two‐ratios model, which assumes different evolutionary patterns between wild boars from Vietnam and Siberia, was compared with one‐ratio model, which assumes the same pattern for wild boars. For VS_wf_, only phylogeny‐based Ka/Ks ratios were determined to avoid sample size bias. Eight models were constructed to include major grouping possibilities (Figures 4 and 5). The null model was “one‐ratio model (1ω),” which assumes that all branches share the same ω ratio. The alternative models were the other six models and “free‐ratio model (Free ω, or Fω)” (Table 1). These models were then ranked by Akaike information criterion (AIC) and Bayesian information criterion (BIC) to select the best model. Likelihood‐ratio test (LRT) was used to determine the significance level for the best model determined by AIC and BIC.

**Table 1:** Tests of the differences in Ka/Ks (or ω) between Vietnamese and Siberian lineages (the methods to categorize branches are explained in Figure 4)

Model	Categories	AIC	BIC	*p* value (LRT)
1ω		30,396.04	15,343.73	
2ω1	Internal(I)/Terminal(T)	30,375.82	15,336.65	2.46E−06 versus 1ω
2ω2	Siberian(S)/Vietnam(V)	30,284.88	15,291.18	≪0.001 versus 1ω
2ω3	Domestic(D)/Wild(W)	30,331.64	15,314.56	4.44E−16 versus 1ω
3ω1	I/ST/VT	30,286.36	15,294.96	≪0.001 versus 2ω1
3ω2	S/VW/VD	30,544.26	15,423.91	≪0.001 versus 2ω2
				≪0.001 versus 1ω
4ω	I/ST/VDT/VWT	30,352.34	15,330.99	1.22E−15
FRM (free ω)		30,354.5	15,459.56	1ω versus 2ω2

In order to evaluate whether the patterns observed from our samples have a general basis, we downloaded and analyzed mitochondrial *Cytb* (cytochrome b) gene sequences. This gene was chosen because it had the richest published sequences within all mitochondrial coding genes for wild boars. In total, the dataset contained 61 and 64 sequences of wild boars (including the 24 sequences assembled from NGS data) from northern and southern Asia, respectively (Supporting information Table S15). The northern group included sequences from subarctic regions of Siberia (Primorsky Krai, Tyva, Zabaykalsky Krai, and Novosibirsk), Mongolia, Korea, northeast China, and northern China (assembled from NGS reads with >25× depth). The southern group comprised samples from Vietnam, Laos, Southwest China (Yunnan province), Southeast China (Hainan, Fujian, Jiangxi, and Zhejiang provinces). The samples with unlikely phylogenetic status (potential European origin or unusually long branch length) were removed. Ka/Ks ratios were computed by CODEML within each group using haplotype sequences. The Ka/Ks outliers without either nonsynonymous or synonymous sites were removed to avoid the bias caused by sequence dependency. In addition, as the elevated Ka/Ks might be due to either relaxation of purifying selection or positive selection in a few sites, we further assessed the possibility of positive selection using an improved version of branch‐site model (Zhang, Nielsen, & Yang, 2005). Branch‐site model test, accommodating Ka/Ks ratios to vary among branches and amino acid sites simultaneously, is very conservative but has better power to distinguish between the relaxation of purifying selection and positive selection (Shen et al., 2010; Zhang et al., 2005). The branches detected with higher Ka/Ks by the above‐mentioned branch‐model were assigned as foreground branches. LRT was used to compare branch‐site model A with the corresponding null model (“M1a”).

### 2.4 Demographic history

**Figure 6:**
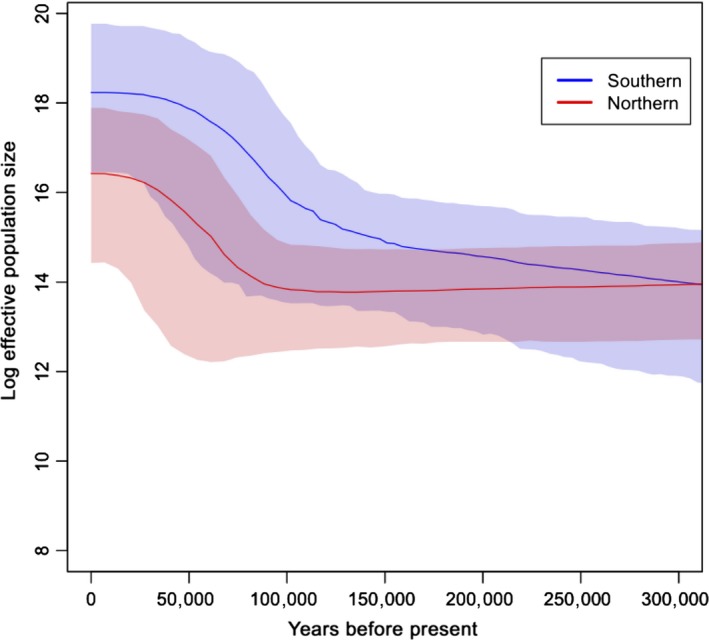
Bayesian skyline plots of wild boars from Siberia and Vietnam as well as nearby regions. The *x*‐axis gives units of years before present, and *y*‐axis is equal to log(4*N*_e_). The shade areas are within the 95% highest posterior density interval

To evaluate the role of *N*_e_ in shaping selection pressure, we employed Bayesian skyline plot (BSP) to infer population history of wild boars (Figure 6). Considering the sensitivity of BSP analysis to sampling numbers, we used D‐loop sequences, which represent the most abundant noncoding marker of mtDNA sequences in NCBI. In total, 408 sequences (including locally assembled NGS data) were incorporated for wild boars from nearby regions of Siberia and Vietnam (160 vs. 248) (Supporting information Table S16). BSPs of *N*_e_ were produced from sequences using MCMC sampling in the program BEAST (version 1.8) (Drummond, Suchard, Xie, & Rambaut, 2012; Ho & Shapiro, 2011). MCMC chain length was set to 10,000,000 with the first 10% of sampled generations discarded as burn‐in to collect sufficient samples for parameter estimation. Piecewise constant model was assumed to generate figures. Estimates of *N*_e_ were derived from the Bayesian coalescent rate through time. Generation time and mutation rate were fixed to 5 years and 2.5 × 10^−8^ substitutions/site/year, respectively, based on previous reports (Bosse et al., 2012; Groenen et al., 2012). Tracer v1.6 was used to determine convergence of model parameters with ESS values (>200), after multiple times of independent MCMC simulations (Rambaut, Suchard, Xie, & Drummond, 2015).

## 3 RESULTS

### 3.1 Phylogenetic structure

At present, complete mitochondrial DNA sequences are scant for both Vietnamese and Siberian wild boars and local domestic pigs. There are only two mitogenome sequences available for Vietnamese wild boar and local pig (GenBank: KU556691.1, Tran et al., 2016; GenBank: EF545584.1, Wu et al., 2007), but no complete mitogenome is available for wild boar from the major region of Siberia (especially the middle region). In this study, complete mitogenome sequences were determined for eight Vietnamese free‐range domestic pigs, nine Vietnamese wild boars, and seven Siberian wild boars. For these 24 samples, 24 haplotypes were detected, suggesting all samples had different maternal backgrounds. The previously unknown phylogenetic positions of Sw and Vwf were inferred from phylogenetic analyses based on three methods (ML, NJ, and BI) (Figure 2c; Supporting information Figures S1 and S2). Consistent with previous studies (Groenen et al., 2012; Wu et al., 2007), all of these inferred trees revealed two major clades, representing wild boars and domestic pigs from Europe and Asia. Despite this divergence between European and Asian clades, there were some European commercial breeds, such as Berkshire, Yorkshire, Landrace, and Large White, nested into the Asian clade. In all trees, both Vietnamese and Siberian samples clustered into Asian clade, but they were characterized by different phylogenetic structures. Siberian wild boars converged into a single subclade (Figure 2c, subclade K), while Vietnamese wild boars and a local breed intermingled with other subclades. The unique evolutionary clustering of Siberian wild boars might suggest their population‐specific migration route (Figure 2a). In an interesting manner, Sw formed a robustly supported clade with wild boar from the Altai mountain region (GenBank: KU057358) (NJ: bootstrap value >0.95), probably due to their relatively small geographic distance. Likewise, Vwf also demonstrated very close relationship with wild boars and local pigs from nearby regions. In all trees, Vietnamese wild boars formed seven subclades with wild boars and domestic pigs from Southwest and South China (Sichuan, Yunnan, Guizhou, Hunan, and Hainan provinces; subclades A, B, C, E, F, M, and M). Vietnamese local pigs clustered within four subclades with wild boars and local pigs in South and Southwest China, including Longlin pigs, Luchuan pigs, and Bama miniature pigs in Guangxi province, Lingao pigs and Wuzhishan pigs in Hainan province, and Qianshao spotted pigs in Guizhou province (subclades E, F, M, and N). Three subclades included both Mongcai pigs and wild boars (subclades A, B, and E). The median‐joining network also supported these different phylogenetic structuring between Siberian wild boars and Vietnamese wild boars and free‐range local pigs (Figure 3).

### 3.2 Ka/Ks of mitogenomes of Sw and Vwf

Both pairwise Ka/Ks and phylogeny‐based Ka/Ks showed similar trend between Siberian and Vietnamese branches. For VS_w_ dataset (Vietnamese and Siberian wild boars), average Ka/Ks ratio (or ω) of Siberian wild boars was lower than that of Vietnamese wild boars (0.137 vs. 0.148). The LRT revealed that two‐ratios model was significantly different from one‐ratio model (LRT *p* < 0.001). Ka/Ks ratios determined by two‐ratios model showed that Siberian wild boars had lower Ka/Ks than Vietnamese wild boars (0.083 vs. 0.106), indicating that Siberian wild boars have accumulated weakly deleterious mutations significantly slower than Vietnamese wild boars. In addition, we observed similar pattern in VS_wf_ dataset, which incorporated free‐range local pigs into VS_w_. Although BIC penalizes model complexity more heavily than AIC (Yang, 2006), both of these criteria revealed that model “2ω2” was the best model for 12 concatenated genes of mitogenomes. This model was also significantly different from the “1ω” model (*p* < 0.001), suggesting that Siberian wild boars evolved differently from Vietnamese wild boars and free‐range pigs. In the “2ω2” model (Siberia vs. Vietnam), the Siberian branches had a significantly lower Ka/Ks than the Vietnamese branches (0.111 vs. 0.184, LRT *p* < 0.001) (Figure 5). It is worth noting that from the datasets VS_w_ to VS_wf_, Ka/Ks of Siberian wild boars increased 1.34 times, while Vietnamese branches increased 1.74 times, indicating that incorporating the sequences of free‐range pigs may inflate Ka/Ks.

We further applied Ka/Ks calculation in more samples of wild boars located in broader regions of southern (five provinces in southern China and two countries in Southeast Asia) and northern Asia (three provinces in northern China, four regions in Siberia, Mongolia, and South Korea). The result showed that both average (0.166 vs. 0.172) and median (0.092 vs. 0.123) of Ka/Ks were higher in southern group than in northern group, demonstrating a similar pattern with the VS_w_ and VS_wf_ datasets. Moreover, as Vietnamese clades had higher Ka/Ks than Siberian clade, we further determined whether this inflation was caused by positive selection using the modified version of branch‐site model (Zhang et al., 2005). Model comparison showed that there was no significant improvement in likelihood when branch‐site “model A” allowed positive selection in a few sits compared with the null model “M1a” (12 genes: lnL, −14856.779 vs. −14858.669, Δ*p* = 1, χ^2^ test, *p *> 0.05; *Cytb*: lnL, −1885.219 vs. −1886.407, Δ*p* = 1, χ^2^ test, *p *> 0.05).

### 3.3 Ka/Ks ratios for each mitochondrial gene

Model selection and LRT were also performed for each mitochondrial gene independently. In an interesting manner, among 12 mitochondrial genes, *Cytb* gene was detected significant difference between Siberian and Vietnamese clades (LRT *p *= 0.04) (Supporting information Additional file 2). For *Cytb* gene, Siberian wild boars had 2.059 times lower Ka/Ks than Vietnamese wild boars and free‐range pigs (0.085 vs. 0.176).

### 3.4 Smaller *N*_e_ in southern wild boars than northern wild boars

According to the corollary of population genetics, the lowered effective population size (*N*_e_) may reduce the efficiency of purifying selection (Kimura, 1985), thus boosting the Ka/Ks ratios up. In other words, the values of *N*_e_ and Ka/Ks might be negatively correlated. As we found that Siberian wild boars had lower Ka/Ks than Vietnamese wild boars, it is very interesting to evaluate whether this reduced Ka/Ks ratio in Siberian wild boars might come from higher *N*_e_ in Siberian wild boars. *N*_e_ is notoriously difficult to quantify in natural populations because the related demographic parameters are usually dynamic in the different time frames. To increase the power of demographic inference, we incorporated public data of wild boars from nearby regions of Siberia and Vietnam (including Sanger sequencing and local assembly of NGS data, Supporting information Table S16). The Bayesian skyline plot (BSP) of 408 D‐loop sequences showed that southern wild boars may have larger *N*_e_ than that of northern wild boars (Figure 6). Although overlap exists between the 95% HPD (highest posterior density) upper limits of northern wild boars and the 95% HPD lower limits of southern wild boars, probably due to the limited resolution of mtDNA compared with whole‐genome data, the overall pattern of this maternal BSP inference is consistent with previous results based on genomewide data (Li et al., 2013). In addition, the lower *N*_e_ in northern wild boars than in southern wild boars was compatible with the expectation based on population migration. It is well known that the early migration of wild boars was initiated from Island Southeast Asia (Frantz et al., 2013; Wu et al., 2007). During this northward range expansion, because of the founder effect, populations may gradually decrease in *N*_e_ with distance from the places of origin (Henn et al., 2016).

## 4 DISCUSSION

There is an ongoing debate on how the accumulation of slightly deleterious mutation might be affected by effective population size (*N*_e_) and selection coefficient. Studies based on population genetics have revealed that along the routes of range expansion, geographically isolated populations often exhibit increased ratios of deleterious mutations as a result of steadily decreasing *N*_e_ with distance from the area of origin (Henn et al., 2016; Peischl, Dupanloup, Kirkpatrick, & Excoffier, 2013). However, ratios of deleterious mutations can also decrease when selection coefficient changes. It has been reported that cold‐water and fast‐moving fish may have reduced ratios of deleterious mutations in mitochondrial genes probably due to stronger selective constraints (Strohm, Gwiazdowski, & Hanner, 2015; Sun et al., 2011). Thus, it is important to resolve the different implications based on demography and selective constraints. We first determined the phylogenetic relationship among Eurasian wild boars and local pigs, then estimated selection pressures based on rigorous statistical approaches, and finally evaluated the potential roles of *N*_e_ in influencing the efficiency of selection.

For Vietnamese samples, phylogenetic and network inference assigned nine Vietnamese wild boars and eight Vietnamese local pigs into seven and four subclades, respectively. The result is compatible with the likely scenario that Vietnam as one of the most important corridors through which the gene flow between wild boars and local pigs from nearby regions may exist. In contrast, Siberian wild boars with long sampling distances (> 1,100 km) were found to cluster into a unique subclade, suggesting the possibility of long‐range contact. Future sequencing of more samples in northern China and Asia may provide better insight into the migration history and pattern of gene flow for wild boars in Eurasia.

The disparate phylogenetic structures of Siberian and Vietnamese samples may indicate their different patterns of molecular evolution. LRT and nested model comparisons reveal the best model as the different evolutionary patterns between samples from Vietnam and Siberia. This pattern still holds even after incorporating samples of Vietnamese free‐range local pigs (Figure 5), suggesting that the differences may not be a coincidence. The Ka/Ks ratios computed by different methods consistently show that Siberian wild boars have significantly lower values than Vietnamese samples. Although the positive selection in a few sites may inflate overall Ka/Ks in mitochondrial genes (Zhang et al., 2005), we fail to detect significant signals of positive selection among amino acid sites of Vietnamese clades with the powerful branch‐site model, suggesting that the comparatively higher Ka/Ks in Vietnamese wild boars may not be attributed to direct positive selection. These results confirm the previous reports that mitochondrial genes are under highly effective purifying selection in mammalian genomes to remove de novo nonsynonymous mutations (Popadin, Nikolaev, Junier, Baranova, & Antonarakis, 2012; Stewart et al., 2008). In an interesting manner, using more sequences of *Cytb* gene from southern and northern latitudes of Asia, we find that northern wild boars have lower Ka/Ks than southern wild boars. This observation is compatible with the results between Siberian and Vietnamese samples, suggesting a potential general basis for the lower Ka/Ks in wild boars of cold regions.

The most parsimonious explanation for the differences in Ka/Ks between Siberian wild boars and Vietnamese samples could be the disparate climates. The two regions have significantly different temperature dynamics in the whole year. For Siberian wild boars, which can survive the cold subarctic climate, energy‐related genes might contribute to their local adaptation. As mitochondria are the powerhouses of cells, which play essential roles in both thermogenesis and thermoregulation (Gambert & Ricquier, 2007; Onda et al., 2008; Simonyan & Skulachev, 1998), mitogenomes of Siberian wild boars may be under stronger purifying constraints than those of tropical wild boars, to avoid the accumulation of nonsynonymous mutations and to keep the stability of mitochondrial function. In addition, compared with animals in tropical areas, animals in cold regions may have to produce more total thermal energy to sustain chilling‐resistance activities such as shaking and shivering (Brück, Wünnenberg, Gallmeier, & Ziehm, 1970; Wei, 1981). Factors including predators and limited food resources could drive them to spend more kinetic energy for foraging activities. Therefore, these stringent selective constraints in cold conditions may be an important agent in driving the evolution of mitogenomes in Siberian wild boars.

Apart from selective constraints, *N*_e_ is another important factor that may influence the accumulation of slightly deleterious mutations. Ample evidence has shown that *N*_e_ might also influence the efficiency of purifying selection and cause changes in proportions of slightly deleterious mutations (Cruz, Vilà, & Webster, 2008; Henn et al., 2016; Kimura, 1962; Schubert et al., 2014). For example, rodents (mouse–rat) may have 1.5 times lower Ka/Ks compared with primates (human–orangutan), probably due to the more effective purifying selection in rodents endowed by their larger *N*_e_. Domesticated animals, compared their wild relatives, have accumulated more nonsynonymous mutations in both nuclear and mitochondrial genomes, probably caused by the reduced selective efficiency following domestication bottleneck and the relaxation of selective constraints in human‐mediated environment (Chen et al., 2018; Cruz et al., 2008). If the selection coefficients remain unchanged, a larger *N*_e_ can increase the efficacy of purifying selection to remove the slightly deleterious mutations, thus resulting in lower Ka/Ks ratios. Based on this rationale, the lower Ka/Ks ratio detected in Siberian wild boars (Figure 5) might also be attributed to their relatively higher *N*_e_. However, this possibility can be eliminated by their migration history, living conditions, our BSP result, and previous whole‐genome results (Li et al., 2013). As one of the most widespread animals, wild boars have adapted to many different environments across Eurasia after migrating out of Southeast Asia millions of years ago (Frantz et al., 2013; Rothschild & Ruvinsky, 2011). The process of long‐distance migration can lead to the reduction in *N*_e_ in regions remote from the place of origin due to the bottleneck effect (Li & Durbin, 2011). In human, non‐African populations are found to have lower *N*_e_ than native African populations (Li & Durbin, 2011; Tenesa et al., 2007). In this sense, considering the migration history of *Sus scrofa*, it seems improbable that Siberian wild boars may have higher *N*_e_ than Vietnamese wild boars. Other factors such as harsh environment and food shortage can also lead to lower *N*_e_ in Siberian wild boars than in tropical wild boars. In particular, considering relatively longer generation times in cold‐region animals (Maiti & Maiti, 2011), the *N*_e_ of Siberian wild boars might be even smaller. Moreover, previous reports and our BSP analysis also reveal that wild boars in northern Asia have lower *N*_e_ than those in southern Asia (Li et al., 2013). Therefore, the lower Ka/Ks ratio in Siberian wild boars should be attributed to their more stringent selective constraints rather than changes in *N*_e_.

Recent studies have emphasized the prominent role of functional constraints in shaping the evolution of mitochondrial genes (Radzvilavicius, Kokko, & Christie, 2017; Strohm et al., 2015; Tavares & Seuánez, 2017). In contrast to the expectation of lowered selective efficiency of the mitogenome due to its much lower *N*_e_ than nuclear genes (~1/4), mitochondrial genes are under much more effective purifying selection than genome‐wide essential genes (Nabholz, Ellegren, & Wolf, 2012). Comparative genomics analyses find that mitochondrial genes have 5.3 times higher selection coefficient than nuclear essential genes (Popadin et al., 2012). This unexpected high selection coefficient is probably due to the high levels of gene expressions of mitochondrial genes (Nabholz et al., 2012). In this study, we find relatively lower Ka/Ks ratios for mitochondrial genes in wild boars from subarctic regions. In particular, the *Cytb* gene has been found to have two times lower Ka/Ks ratio in Siberian wild boars than in Vietnamese wild boars (0.085 vs. 0.1758, LRT *p *= 0.04). This difference may imply that stronger selective constraints have imposed on *Cytb* gene of wild boars from cold regions to sustain its important functions. It has been reported that *Cytb* gene may play a more important role in energy production than in heat generation (Sun et al., 2011). The stable ability to produce energy could be very important for Siberian wild boars to forage for food over long distances. This possibility is also supported by the close relationship (Figure 2c, subclade K) between wild boars from three distant localities in Siberia. The functional importance of cytochrome b in producing energy for long‐range foraging might facilitate the close relationship among Siberian wild boars.

## 5 CONCLUSIONS

The mitogenomes of wild boars (and free‐range local pigs) from tropical and cold regions (Vietnam and Siberia) have been sequenced to infer their phylogenetic status, selection pressures, and historical demography. Although from three localities, Siberian wild boars are phylogenetically grouped into a single subclade, suggesting their monophyletic origin. Both the pairwise Ka/Ks and the best model determined by model selection with associated LRT show that Siberian wild boars have a significantly lower Ka/Ks than Vietnamese wild boars and free‐range local pigs, suggesting stronger selective constraints in mitogenomes of Siberian wild boars. An alternative scenario is that the positive selection in only a few sites might account for the higher Ka/Ks in Vietnamese samples. However, this possibility gains little credence from result of branch‐site model analysis in that there is no significant improvement in likelihood when allowing a few sites to evolve under positive selection for Vietnamese clades. Moreover, by extending Ka/Ks calculation into wild boars from more broader regions of southern and northern Asia, we find relatively lower Ka/Ks for northern wild boars, suggesting a more general trend of stronger selective constraints in energy‐related mitochondrial genes for wild boars from cold regions. Factors including migration history, food shortage, cold climatic conditions, and demographic analyses have ruled out the possible role of *N*_e_ in decreasing the Ka/Ks ratio of Siberian wild boars, as it seems improbable that Siberian wild boars could have higher *N*_e_ than Vietnamese wild boars. In an interesting manner, *Cytb* gene shows significantly lower Ka/Ks ratios in Siberian wild boars than in Vietnamese wild boars and free‐range local pigs, suggesting the important role of cytochrome b for adaptions to cold conditions. This study demonstrates empirical evidence in which the effect of selective constraints might be stronger than *N*_e_ in causing the slower accumulation of slightly deleterious mutations.

## CONFLICT OF INTEREST

The authors report that no conflict of interests exist.

## AUTHOR CONTRIBUTION

JHC, SHZ, and JLH conceived the ideas and designed the research; JHC, PN, EVK, VLP, XDL, and TNTT collected and analyzed data. JHC led the writing of the manuscript with substantial contribution from NŠ and JLH; all authors commented on the manuscript.

## DATA ACCESSIBILITY

DNA sequences: GenBank (NCBI) accessions KX982629–KX982652.

## References

[ece34221-bib-0001] Angelis, K. , dos Reis, M. , & Yang, Z. (2014). Bayesian estimation of nonsynonymous/synonymous rate ratios for pairwise sequence comparisons. Molecular Biology and Evolution, 31(7), 1902–1913. 10.1093/molbev/msu142 24748652 PMC4069626

[ece34221-bib-0002] Bandelt, H.‐J. , Forster, P. , & Röhl, A. (1999). Median‐joining networks for inferring intraspecific phylogenies. Molecular Biology and Evolution, 16(1), 37–48. 10.1093/oxfordjournals.molbev.a026036 10331250

[ece34221-bib-0003] Björnerfeldt, S. , Webster, M. T. , & Vilà, C. (2006). Relaxation of selective constraint on dog mitochondrial DNA following domestication. Genome Research, 16(8), 990–994. 10.1101/gr.5117706 16809672 PMC1524871

[ece34221-bib-0004] Block, B. A. (1994). Thermogenesis in muscle. Annual Review of Physiology, 56(1), 535–577. 10.1146/annurev.ph.56.030194.002535 8010751

[ece34221-bib-0005] Bolger, A. M. , Lohse, M. , & Usadel, B. (2014). Trimmomatic: A flexible trimmer for Illumina sequence data. Bioinformatics, 30(15), 2114–2120. 10.1093/bioinformatics/btu170 24695404 PMC4103590

[ece34221-bib-0006] Bosse, M. , Megens, H.‐J. , Madsen, O. , Paudel, Y. , Frantz, L. A. F. , Schook, L. B. , … Groenen, M. A. (2012). Regions of homozygosity in the porcine genome: Consequence of demography and the recombination landscape. PLoS Genetics, 8(11), e1003100 10.1371/journal.pgen.1003100 23209444 PMC3510040

[ece34221-bib-0007] Brück, K. , Wünnenberg, W. , Gallmeier, H. , & Ziehm, B. (1970). Shift of threshold temperature for shivering and heat polypnea as a mode of thermal adaptation. Pflügers Archiv—European Journal of Physiology, 321(2), 159–172. 10.1007/BF00586370 5529538

[ece34221-bib-0008] Chen, J. , Ni, P. , Li, X. , Han, J. , Jakovlić, I. , Zhang, C. , & Zhao, S. (2018). Population size may shape the accumulation of functional mutations following domestication. BMC Evolutionary Biology, 18(1), 4 10.1186/s12862-018-1120-6 29351740 PMC5775542

[ece34221-bib-0009] Cruz, F. , Vilà, C. , & Webster, M. T. (2008). The legacy of domestication: Accumulation of deleterious mutations in the dog genome. Molecular Biology and Evolution, 25(11), 2331–2336. 10.1093/molbev/msn177 18689870

[ece34221-bib-0010] Darriba, D. , Taboada, G. L. , Doallo, R. , & Posada, D. (2012). jModelTest 2: More models, new heuristics and parallel computing. Nature Methods, 9(8), 772 10.1038/nmeth.2109 PMC4594756 22847109

[ece34221-bib-0011] Drummond, A. J. , Suchard, M. A. , Xie, D. , & Rambaut, A. (2012). Bayesian phylogenetics with BEAUti and the BEAST 1.7. Molecular Biology and Evolution, 29(8), 1969–1973. 10.1093/molbev/mss075 22367748 PMC3408070

[ece34221-bib-0012] Frantz, L. A. F. , Schraiber, J. G. , Madsen, O. , Megens, H.‐J. , Bosse, M. , Paudel, Y. , … Groenen, M. A. M. (2013). Genome sequencing reveals fine scale diversification and reticulation history during speciation in *Sus* . Genome Biology, 14(9), R107 10.1186/gb-2013-14-9-r107 24070215 PMC4053821

[ece34221-bib-0013] Gambert, S. , & Ricquier, D. (2007). Mitochondrial thermogenesis and obesity. Current Opinion in Clinical Nutrition & Metabolic Care, 10(6), 664–670. 10.1097/MCO.0b013e3282f0b69d 18089945

[ece34221-bib-0014] Groenen, M. A. M. , Archibald, A. L. , Uenishi, H. , Tuggle, C. K. , Takeuchi, Y. , Rothschild, M. F. , … Schook, L. B. (2012). Analyses of pig genomes provide insight into porcine demography and evolution. Nature, 491(7424), 393–398. 10.1038/nature11622 23151582 PMC3566564

[ece34221-bib-0015] Hard, D. , & Clark, A. (1989). Principles of population genetics. Sunderland, MA: Sinauer Associates, Inc., Publishers.

[ece34221-bib-0016] Hasegawa, M. , Cao, Y. , & Yang, Z. (1998). Preponderance of slightly deleterious polymorphism in mitochondrial DNA: Nonsynonymous/synonymous rate ratio is much higher within species than between species. Molecular Biology and Evolution, 15(11), 1499–1505. 10.1093/oxfordjournals.molbev.a025877 12572613

[ece34221-bib-0017] Henn, B. M. , Botigue, L. R. , Peischl, S. , Dupanloup, I. , Lipatov, M. , Maples, B. K. , … Bustamante, C. D. (2016). Distance from sub‐Saharan Africa predicts mutational load in diverse human genomes. Proceedings of the National Academy of Sciences of the United States of America, 113(4), E440–E449. 10.1073/pnas.1510805112 26712023 PMC4743782

[ece34221-bib-0018] Ho, S. Y. W. , & Shapiro, B. (2011). Skyline‐plot methods for estimating demographic history from nucleotide sequences. Molecular Ecology Resources, 11(3), 423–434. 10.1111/j.1755-0998.2011.02988.x 21481200

[ece34221-bib-0019] Hughes, A. L. (2013). Accumulation of slightly deleterious mutations in the mitochondrial genome: A hallmark of animal domestication. Gene, 515(1), 28–33. 10.1016/j.gene.2012.11.064 23237775 PMC3895481

[ece34221-bib-0020] Katoh, K. , Misawa, K. , Kuma, K. I. , & Miyata, T. (2002). MAFFT: A novel method for rapid multiple sequence alignment based on fast Fourier transform. Nucleic Acids Research, 30(14), 3059–3066. 10.1093/nar/gkf436 12136088 PMC135756

[ece34221-bib-0021] Kimura, M. (1962). On the probability of fixation of mutant genes in a population. Genetics, 47(6), 713–719. https://www.ncbi.nlm.nih.gov/pmc/articles/PMC1210364/pdf/713.pdf 14456043 10.1093/genetics/47.6.713 PMC1210364

[ece34221-bib-0022] Kimura, M. (1985). The neutral theory of molecular evolution. Cambridge: Cambridge University Press.

[ece34221-bib-0023] Kryukov, G. V. , Pennacchio, L. A. , & Sunyaev, S. R. (2007). Most rare missense alleles are deleterious in humans: Implications for complex disease and association studies. The American Journal of Human Genetics, 80(4), 727–739. 10.1086/513473 17357078 PMC1852724

[ece34221-bib-0024] Leigh, J. W. , & Bryant, D. (2015). PopART: Full‐feature software for haplotype network construction. Methods in Ecology and Evolution, 6(9), 1110–1116. 10.1111/2041-210X.12410

[ece34221-bib-0025] Li, H. , & Durbin, R. (2011). Inference of human population history from individual whole‐genome sequences. Nature, 475(7357), 493–496. 10.1038/nature10231 21753753 PMC3154645

[ece34221-bib-0026] Li, M. , Tian, S. , Jin, L. , Zhou, G. , Li, Y. , Zhang, Y. , … Li, R. (2013). Genomic analyses identify distinct patterns of selection in domesticated pigs and Tibetan wild boars. Nature Genetics, 45(12), 1431 10.1038/ng.2811 24162736

[ece34221-bib-0027] Librado, P. , & Rozas, J. (2009). DnaSP v5: A software for comprehensive analysis of DNA polymorphism data. Bioinformatics, 25(11), 1451–1452. 10.1093/bioinformatics/btp187 19346325

[ece34221-bib-0028] Maiti, P. K. , & Maiti, P. (2011). Biodiversity: Perception, peril and preservation. Delhi: Prentice‐Hall of India Pvt. Ltd.

[ece34221-bib-0029] McGinnis, S. , & Madden, T. L. (2004). BLAST: At the core of a powerful and diverse set of sequence analysis tools. Nucleic Acids Research, 32(Suppl. 2), W20–W25. 10.1093/nar/gkh435 15215342 PMC441573

[ece34221-bib-0030] Mezmouk, S. , & Ross‐Ibarra, J. (2014). The pattern and distribution of deleterious mutations in maize. G3: Genes|Genomes|Genetics, 4(1), 163–171. 10.1534/g3.113.008870 24281428 PMC3887532

[ece34221-bib-0031] Mitterboeck, T. F. , & Adamowicz, S. J. (2013). Flight loss linked to faster molecular evolution in insects. Proceedings of the Royal Society of London B: Biological Sciences, 280(1767), 20131128 10.1098/rspb.2013.1128 PMC3735250 23884090

[ece34221-bib-0032] Nabholz, B. , Ellegren, H. , & Wolf, J. B. (2012). High levels of gene expression explain the strong evolutionary constraint of mitochondrial protein‐coding genes. Molecular Biology and Evolution, 30(2), 272–284. 10.1093/molbev/mss238 23071102

[ece34221-bib-0033] Ohta, T. (1973). Slightly deleterious mutant substitutions in evolution. Nature, 246(5428), 96–98. 10.1038/246096a0 4585855

[ece34221-bib-0034] Onda, Y. , Kato, Y. , Abe, Y. , Ito, T. , Morohashi, M. , Ito, Y. , … Ito, K. (2008). Functional coexpression of the mitochondrial alternative oxidase and uncoupling protein underlies thermoregulation in the thermogenic florets of skunk cabbage. Plant Physiology, 146(2), 636–645. 10.1104/pp.107.113563 18162588 PMC2245847

[ece34221-bib-0035] Peischl, S. , Dupanloup, I. , Kirkpatrick, M. , & Excoffier, L. (2013). On the accumulation of deleterious mutations during range expansions. Molecular Ecology, 22(24), 5972–5982. 10.1111/mec.12524 24102784

[ece34221-bib-0036] Popadin, K. Y. , Nikolaev, S. I. , Junier, T. , Baranova, M. , & Antonarakis, S. E. (2012). Purifying selection in mammalian mitochondrial protein‐coding genes is highly effective and congruent with evolution of nuclear genes. Molecular Biology and Evolution, 30(2), 347–355. 10.1093/molbev/mss219 22983951

[ece34221-bib-0037] Radzvilavicius, A. L. , Kokko, H. , & Christie, J. R. (2017). Mitigating mitochondrial genome erosion without recombination. Genetics, 207(3), 1079–1088. 10.1534/genetics.117.300273 28893855 PMC5676227

[ece34221-bib-0038] Rambaut, A. , Suchard, M. A. , Xie, D. , & Drummond, A. J. (2015). Tracer v1. 6. Accessed in December 2015. Available from http://tree.bio.ed.ac.uk/

[ece34221-bib-0039] Ronquist, F. , Teslenko, M. , van der Mark, P. , Ayres, D. L. , Darling, A. , Höhna, S. , … Huelsenbeck, J. P. (2012). MrBayes 3.2: Efficient Bayesian phylogenetic inference and model choice across a large model space. Systematic Biology, 61(3), 539–542. 10.1093/sysbio/sys029 22357727 PMC3329765

[ece34221-bib-0040] Rothschild, M. F. , & Ruvinsky, A. (2011). The genetics of the pig. Wallingford: CABI 10.1079/9781845937560.0000

[ece34221-bib-0041] Rowland, L. A. , Bal, N. C. , & Periasamy, M. (2015). The role of skeletal‐muscle‐based thermogenic mechanisms in vertebrate endothermy. Biological Reviews, 90(4), 1279–1297. 10.1111/brv.12157 25424279 PMC4854186

[ece34221-bib-0042] Schubert, M. , Jónsson, H. , Chang, D. , Der Sarkissian, C. , Ermini, L. , Ginolhac, A. , … Orlando, L. (2014). Prehistoric genomes reveal the genetic foundation and cost of horse domestication. Proceedings of the National Academy of Sciences of the United States of America, 111(52), E5661–E5669. 10.1073/pnas.1416991111 25512547 PMC4284583

[ece34221-bib-0043] Shen, Y.‐Y. , Liang, L. , Zhu, Z.‐H. , Zhou, W.‐P. , Irwin, D. M. , & Zhang, Y.‐P. (2010). Adaptive evolution of energy metabolism genes and the origin of flight in bats. Proceedings of the National Academy of Sciences of the United States of America, 107(19), 8666–8671. 10.1073/pnas.0912613107 20421465 PMC2889356

[ece34221-bib-0044] Shen, Y.‐Y. , Shi, P. , Sun, Y.‐B. , & Zhang, Y.‐P. (2009). Relaxation of selective constraints on avian mitochondrial DNA following the degeneration of flight ability. Genome Research, 19(10), 1760–1765. 10.1101/gr.093138.109 19617397 PMC2765268

[ece34221-bib-0045] Simonyan, R. A. , & Skulachev, V. P. (1998). Thermoregulatory uncoupling in heart muscle mitochondria: Involvement of the ATP/ADP antiporter and uncoupling protein. FEBS Letters, 436(1), 81–84. 10.1016/S0014-5793(98)01106-5 9771898

[ece34221-bib-0046] Skulachev, V. P. (1999). Mitochondrial physiology and pathology; concepts of programmed death of organelles, cells and organisms. Molecular Aspects of Medicine, 20(3), 139–184. 10.1016/S0098-2997(99)00008-4 10626278

[ece34221-bib-0047] Stamatakis, A. (2014). RAxML version 8: A tool for phylogenetic analysis and post‐analysis of large phylogenies. Bioinformatics, 30(9), 1312–1313. 10.1093/bioinformatics/btu033 24451623 PMC3998144

[ece34221-bib-0048] Stewart, J. B. , Freyer, C. , Elson, J. L. , & Larsson, N.‐G. (2008). Purifying selection of mtDNA and its implications for understanding evolution and mitochondrial disease. Nature Reviews Genetics, 9(9), 657–662. 10.1038/nrg2396 18695671

[ece34221-bib-0049] Strohm, J. H. , Gwiazdowski, R. A. , & Hanner, R. (2015). Fast fish face fewer mitochondrial mutations: Patterns of dN/dS across fish mitogenomes. Gene, 572(1), 27–34. 10.1016/j.gene.2015.06.074 26149654

[ece34221-bib-0050] Sun, Y.‐B. , Shen, Y.‐Y. , Irwin, D. M. , & Zhang, Y.‐P. (2011). Evaluating the roles of energetic functional constraints on teleost mitochondrial‐encoded protein evolution. Molecular Biology and Evolution, 28(1), 39–44. 10.1093/molbev/msq256 20924083

[ece34221-bib-0051] Tamura, K. , Stecher, G. , Peterson, D. , Filipski, A. , & Kumar, S. (2013). MEGA6: Molecular evolutionary genetics analysis version 6.0. Molecular Biology and Evolution, 30(12), 2725–2729. 10.1093/molbev/mst197 24132122 PMC3840312

[ece34221-bib-0052] Tavares, W. C. , & Seuánez, H. N. (2017). Disease‐associated mitochondrial mutations and the evolution of primate mitogenomes. PLoS ONE, 12(5), e0177403 10.1371/journal.pone.0177403 28510580 PMC5433710

[ece34221-bib-0053] Tenesa, A. , Navarro, P. , Hayes, B. J. , Duffy, D. L. , Clarke, G. M. , Goddard, M. E. , & Visscher, P. M. (2007). Recent human effective population size estimated from linkage disequilibrium. Genome Research, 17(4), 520–526. 10.1101/gr.6023607 17351134 PMC1832099

[ece34221-bib-0054] Tran, T. N. T. , Ni, P. , Chen, J. , Le, T. T. , Steve, K. , Han, J. , … Zhao, S. (2016). The complete mitochondrial genome of Mong Cai pig (*Sus scrofa*) in Vietnam. Mitochondrial DNA Part B, 1(1), 226–227. 10.1080/23802359.2016.1155424 PMC7800876 33473460

[ece34221-bib-0055] Wang, Z. , Yonezawa, T. , Liu, B. , Ma, T. , Shen, X. , Su, J. , … Liu, J. (2011). Domestication relaxed selective constraints on the yak mitochondrial genome. Molecular Biology and Evolution, 28(5), 1553–1556. 10.1093/molbev/msq336 21156878

[ece34221-bib-0056] Wei, E. T. (1981). Pharmacological aspects of shaking behavior produced by TRH, AG‐3‐5, and morphine withdrawal. Federation Proceedings, 40(5), 1491–1496. https://www.ncbi.nlm.nih.gov/pubmed/6260535 6260535

[ece34221-bib-0057] Wu, G.‐S. , Yao, Y.‐G. , Qu, K.‐X. , Ding, Z.‐L. , Li, H. , Palanichamy, M. G. , … Zhang, Y.‐P. (2007). Population phylogenomic analysis of mitochondrial DNA in wild boars and domestic pigs revealed multiple domestication events in East Asia. Genome Biology, 8(11), R245 10.1186/gb-2007-8-11-r245 18021448 PMC2258183

[ece34221-bib-0058] Yang, Z. (2006). Computational molecular evolution. Oxford: Oxford University Press 10.1093/acprof:oso/9780198567028.001.0001

[ece34221-bib-0059] Yang, Z. (2007). PAML 4: Phylogenetic analysis by maximum likelihood. Molecular Biology and Evolution, 24(8), 1586–1591. 10.1093/molbev/msm088 17483113

[ece34221-bib-0060] Zhang, J. , Nielsen, R. , & Yang, Z. (2005). Evaluation of an improved branch‐site likelihood method for detecting positive selection at the molecular level. Molecular Biology and Evolution, 22(12), 2472–2479. 10.1093/molbev/msi237 16107592

