# Supplementary material for: Selective constraints in cold‐region wild boars may defuse the effects of small effective population size on molecular evolution of mitogenomes
Source: Ecol Evol. 2018 Jul 21;8(16):8102–14. doi: 10.1002/ece3.4221 (PMC6144961; doi:10.1002/ece3.4221)
Supplement: Supplementary file 1 [file ECE3-8-8102-s001.docx]

Figure S1. NJ phylogenetic tree of domestic pigs and wild boars from Europe and Asia based on complete mtDNA sequences. The tree was constructed by MEGA6 with an Africa warthog (NCBI: DQ409327) as the outgroup. Bootstrap supports for major clades were based on 1,000 replicates. The taxa of red clade are Siberian wild boars, and blue lineages are Vietnam wild boars and domestic pigs.

Figure S2. Bayesian phylogenetic tree of domestic pigs and wild boars from Europe and Asia based on complete mtDNA sequences. The tree was constructed by Mrbayes v3.2 with an Africa warthog (NCBI: DQ409327) as the outgroup. The taxa of red clade represent Siberian wild boars, and blue lineages represent Vietnam wild boars and domestic pigs. Bootstrap supports for major clades were shown near the nodes.
