# Supplementary material for: Selective constraints in cold‐region wild boars may defuse the effects of small effective population size on molecular evolution of mitogenomes
Source: Ecol Evol. 2018 Jul 21;8(16):8102–14. doi: 10.1002/ece3.4221 (PMC6144961; doi:10.1002/ece3.4221)
Supplement: Supplementary file 2 [file ECE3-8-8102-s002.docx]

**Table S1: Data used for phylogenetic inference.**

| **Accession ID** | **source** | **Location** | **breeds** |
| --- | --- | --- | --- |
| KP294522 | Downloaded | China: Yuexi, Anhui | Dabieshanblack |
| KP223728 | Downloaded | China: Southern Gansu | Jeuma |
| AB298688 | Downloaded | Japan | Ohmini |
| KP126954 | Downloaded | China | Luchuan |
| KC505409 | Downloaded | China | Yanan |
| KC505408 | Downloaded | China | Wujin |
| KC505407 | Downloaded | China | Penzhou |
| KC505406 | Downloaded | China | Neijiang |
| KP765604 | Downloaded | China | Wuhuang |
| KP765603 | Downloaded | China | Chenghua |
| KP126939 | Downloaded | China | Bamaxiang |
| KJ720205 | Downloaded | China | LaiwuBlack |
| KC493609 | Downloaded | China | GannanTibetan |
| GQ220328 | Downloaded | China | BannaMini |
| AF034253 | Downloaded | Europe | Landrace |
| GQ220329 | Downloaded | China | Dahe |
| KF888634 | Downloaded | China | Bamaminiature |
| DQ972936 | Downloaded | China: Taiwan | IILanyu |
| KC493612 | Downloaded | China: Tibetan | ShigatseTibetan |
| KC493611 | Downloaded | China: Tibetan | NyingchiTibetan |
| KC493610 | Downloaded | China: Tibetan | GanziTibetan |
| KC493608 | Downloaded | China: Tibetan | Tibetan |
| KC493607 | Downloaded | China: Tibetan | Tibetan |
| NC000845 | Downloaded | Europe | Landrace |
| KU556691 | Downloaded | Vietnam | MongCai |
| DQ334861 | Downloaded | Korean | Jejunative |
| DQ334860 | Downloaded | Korean | Jejunative |
| KM998967 | Downloaded | China | Meishan |
| AJ002189 | Downloaded |  | not clear |
| KM275217 | Downloaded | China | Luchuan |
| KM200762 | Downloaded | China | Dahuabai |
| KM433673 | Downloaded | China | Longlin |
| KM094194 | Downloaded | China | Sandublack |
| KM250424 | Downloaded | China | Congjiangminiature |
| KM259826 | Downloaded | China | WuyiBlack |
| KM044240 | Downloaded | China | Diannan |
| KM044239 | Downloaded | China | Rongchang |
| KM073256 | Downloaded | China | Tibetan |
| KM101043 | Downloaded | China | Lantang |
| KF767443 | Downloaded | China | Wuzhishan |
| KF752550 | Downloaded | Europe | Yorkshine |
| KF601700 | Downloaded | China | TaoyuanBlack |
| KF472179 | Downloaded | China | Daweizi |
| KF472178 | Downloaded | China | Ningxiang |
| KF472177 | Downloaded | China | Shaziling |
| DQ518915 | Downloaded | China: Taiwan | Lanyu |
| EF375877 | Downloaded | China: Taiwan | ILanyu |
| EU090703 | Downloaded | Korean | Koreanwildboar |
| EU090702 | Downloaded | Korean | Koreanwildboar |
| AY334492 | Downloaded | Korean | Jejunative |
| DQ274110 | Downloaded | Korean | Jejunative |
| DQ268530 | Downloaded | Korean | Koreanwildboar |
| DQ207753 | Downloaded | Korean | Koreanwildboar |
| DQ207754 | Downloaded | Korean | Koreanwildboar |
| AY574048 | Downloaded | Europe | LargeWhite |
| AY574047 | Downloaded |  | not clear |
| AY574046 | Downloaded | Europe | Hampshire |
| AY574045 | Downloaded | Europe | Berkshire |
| AY337045 | Downloaded | Europe | Duroc |
| DQ207755 | Downloaded | Korean | Koreanwildboar |
| KT372134 | Downloaded | Europe | miniY1 |
| KJ737423 | Downloaded | China | Luchuan |
| KJ737422 | Downloaded | China | Longlin |
| KJ737421 | Downloaded | China | Laiwu |
| KJ737420 | Downloaded | China | Huoshoublack |
| KJ737419 | Downloaded | China | Huai |
| KJ737418 | Downloaded | China | Hang |
| KJ737417 | Downloaded | China | DingYuan |
| KC505411 | Downloaded | China: Southwest China | wildboar |
| KC505410 | Downloaded | Europe | Berkshire |
| KF569218 | Downloaded | Europe | DLY |
| KC250275 | Downloaded | Europe | LargeWhite |
| KC250274 | Downloaded | China | Lantang |
| KC250273 | Downloaded | China | Xiang |
| FJ237003 | Downloaded | Europe | EuropeanwildboarWB6 |
| FJ237002 | Downloaded | Europe | EuropeanwildboarWB5 |
| FJ237001 | Downloaded | Europe | EuropeanwildboarWB4 |
| FJ237000 | Downloaded | Europe | EuropeanwildboarWB3 |
| FJ236999 | Downloaded | Europe | EuropeanwildboarWB2 |
| FJ236998 | Downloaded | Europe | EuropeanwildboarWB1 |
| FJ236997 | Downloaded | Europe | DurocD2 |
| FJ236996 | Downloaded | Europe | DurocD1 |
| FJ236995 | Downloaded | Europe | IberianH6 |
| FJ236994 | Downloaded | Europe | IberianH5 |
| FJ236993 | Downloaded | Europe | IberianH4 |
| FJ236992 | Downloaded | Europe | IberianH3 |
| FJ236991 | Downloaded | Europe | IberianH2 |
| DQ534707 | Downloaded | China | Taoyuan |
| EU333163 | Downloaded | China | ChinesenortheastWB |
| EU117375 | Downloaded | Europe | Iberian |
| AF486874 | Downloaded | Europe | LargeWhite |
| AF486873 | Downloaded | China | ChineseWannanhua |
| AF486872 | Downloaded | China | ChineseJiangquhai |
| AF486871 | Downloaded | China | ChineseYushanhei |
| AF486870 | Downloaded | China | ChineseDahuabai |
| AF486869 | Downloaded | China | DiannanShortear |
| AF486868 | Downloaded | China | Yimenghei |
| AF486867 | Downloaded | China | Wuzhishan |
| AF486866 | Downloaded | Europe | Landerace |
| AF486865 | Downloaded | China | ChineseQingping |
| AF486864 | Downloaded | China | ChineseMin |
| AF486863 | Downloaded | China | ChineseJinhua |
| AF486862 | Downloaded | China | ChineseTongcheng |
| AF486861 | Downloaded | China | ChineseErhualian |
| AF486860 | Downloaded | China | ChineseRongchang |
| AF486859 | Downloaded | China | ChineseXiang |
| AF486858 | Downloaded | Europe | Duroc |
| AF486857 | Downloaded | China | ChineseNingxiang |
| AF486856 | Downloaded | China | ChineseTibetan |
| AF486855 | Downloaded | China | ChineseZhongMeishan |
| GQ351599 | Downloaded | Korean | native pigs |
| DQ466081 | Downloaded | China | Nuogu |
| EF545593 | Downloaded | Korean | KoreanNative |
| EF545592 | Downloaded | Malaysia | myw4 |
| EF545591 | Downloaded | China | Bihu199 |
| EF545590 | Downloaded | China | Bihu208 |
| EF545589 | Downloaded | China | yimh42 |
| EF545588 | Downloaded | China | huzu4 |
| EF545587 | Downloaded | China | huzu2 |
| EF545586 | Downloaded | China | Yunnanwb371 |
| EF545585 | Downloaded | China | Yunnanwb326 |
| EF545584 | Downloaded | Vietnam | VietNamWildboar |
| EF545583 | Downloaded | China | Bamei |
| EF545582 | Downloaded | China | Qingping |
| EF545581 | Downloaded | China | Qingping |
| EF545580 | Downloaded | China | JilinWildboar |
| EF545579 | Downloaded | China | JiangXiwb |
| EF545578 | Downloaded | China | Aaba |
| EF545577 | Downloaded | China | weiAnhui |
| EF545576 | Downloaded | China | diqing |
| EF545575 | Downloaded | China | JiangxiShanggao |
| EF545574 | Downloaded | China | saba |
| EF545573 | Downloaded | China | wbYunnan |
| EF545572 | Downloaded | China | wbHainan |
| EF545571 | Downloaded | China | wbFujian |
| EF545570 | Downloaded | China | wb1Fujian |
| EF545569 | Downloaded | China | wbFujian |
| EF545568 | Downloaded | China | wbYunnan |
| EF545567 | Downloaded | China | wbYunnan |
| KT279760 | Downloaded | Australian | Australian |
| KT279759 | Downloaded | Australian | Australian |
| KT279758 | Downloaded | Australian | Australian |
| KT261430 | Downloaded | Australian | Australian |
| KT261429 | Downloaded | Australian | Australian |
| KM101042 | Downloaded | China | Guanling |
| KP765605 | Downloaded | China | ChangbaimountainsWB |
| KP765602 | Downloaded | Europe | Berkshire |
| KP681245 | Downloaded | China | wbHainan |
| KP681244 | Downloaded | China | Wenchang |
| KP681243 | Downloaded | China | Wuzhishan |
| KP681242 | Downloaded | China | Lingao |
| KP257599 | Downloaded | China | Qingyu |
| KP257598 | Downloaded | China | Liangshan |
| KJ782448 | Downloaded | China | NOt clear |
| KJ909516 | Downloaded | China | Wuzhishan |
| JN6010751 | Downloaded | Europe | Yorkshire |
| JN601074 | Downloaded | Europe | Yorkshire |
| JN6010733 | Downloaded | Europe | Turopolje |
| JN6010722 | Downloaded | Europe | Turopolje |
| JN601071 | Downloaded | China | BMeishan |
| JN601070 | Downloaded | China | AMeishan |
| JN601069 | Downloaded | Europe | Mangalitsa |
| JN601068 | Downloaded | Europe | Mangalitsa |
| JN601067 | Downloaded | Europe | Mangalitsa |
| JN601066 | Downloaded | Europe | Mangalitsa |
| KF660222 | Downloaded | China | QianshaoSpotted |
| KC469586 | Downloaded | China | ChineseJinhua |
| AF304203 | Downloaded | Europe | Swedishwildboarpartial |
| AF304202 | Downloaded | Europe | Landracepartial |
| AF304201 | Downloaded | Europe | Italianwildboarpartial |
| AF304200 | Downloaded | China | ChineseMeishanpartial |
| KF767444 | Downloaded | Europe | Duroc |
| KU057358 | Downloaded | China:Xinjiang | Aletaiwild |
| AP003428 | Downloaded | China | domestica |
| KT965278 | Downloaded | China | IndiaDomestic |
| NC014692 | Downloaded | China:Taiwan | taiwanensis |
| KJ746666 | Downloaded | Europe | Mangalica |
| KJ746665 | Downloaded | China | Yacha |
| KJ746664 | Downloaded | China | Penzhoushandi |
| KJ746663 | Downloaded | China | Neijiang |
| KJ746662 | Downloaded | China | Mashen |
| KF971862 | Downloaded | China | Min |
| KC469587 | Downloaded | Europe | pietrain |
| NC012095 | Downloaded | Europe | Largewhite |
| GU147934 | Downloaded | China | taiwanensis |
| KJ789952 | Downloaded | not clear | not clear |
| KP301137 | Downloaded | not clear | not clear |
| RussiaWildBoarCO | This study | Russia | wildboar |
| RussiaWildBoarT1 | This study | Russia | wildboar |
| RussiaWildBoarT2 | This study | Russia | wildboar |
| RussiaWildBoarT3 | This study | Russia | wildboar |
| RussiaWildBoarW1 | This study | Russia | wildboar |
| RussiaWildBoarW2 | This study | Russia | wildboar |
| RussiaWildBoarZK | This study | Russia | wildboar |
| VNwildboarVNF54 | This study | Vietnam | wildboar |
| VNlocalMCA5 | This study | Vietnam | MongCai |
| VNlocalMCA8 | This study | Vietnam | MongCai |
| VNwildboarF06 | This study | Vietnam | wildboar |
| VNwildboarM11 | This study | Vietnam | wildboar |
| VNwildboarM07 | This study | Vietnam | wildboar |
| VNwildboarF22 | This study | Vietnam | wildboar |
| VNwildboarM09 | This study | Vietnam | wildboar |
| VNwildboarF50 | This study | Vietnam | wildboar |
| VNwildboarM03 | This study | Vietnam | wildboar |
| VNwildboarF08 | This study | Vietnam | wildboar |
| VNLocalMCA18 | This study | Vietnam | MongCai |
| VNLocalMCC09 | This study | Vietnam | MongCai |
| VNLocalMCN16 | This study | Vietnam | MongCai |
| VNLocalMCB11 | This study | Vietnam | MongCai |
| VNLocalMCB27 | This study | Vietnam | MongCai |
| VNLocalMCB40 | This study | Vietnam | MongCai |
| NEChineseWB1 | NGS local assembly | North China | Northern_WildBoar |
| NEChineseWB3 | NGS local assembly | North China | Northern_WildBoar |
| NEChineseWB4 | NGS local assembly | North China | Northern_WildBoar |
| KoreanWB1 | NGS local assembly | North China | Northern_WildBoar |
| KoreanWB10 | NGS local assembly | North China | Northern_WildBoar |
| KoreanWB2 | NGS local assembly | North China | Northern_WildBoar |
| KoreanWB3 | NGS local assembly | North China | Northern_WildBoar |
| KoreanWB4 | NGS local assembly | North China | Northern_WildBoar |
| KoreanWB5 | NGS local assembly | North China | Northern_WildBoar |
| KoreanWB6 | NGS local assembly | North China | Northern_WildBoar |
| KoreanWB7 | NGS local assembly | North China | Northern_WildBoar |
| KoreanWB8 | NGS local assembly | North China | Northern_WildBoar |
| KoreanWB9 | NGS local assembly | North China | Northern_WildBoar |
| NChineseWB30U08 | NGS local assembly | North China | Northern_WildBoar |
| NChineseWB30U09 | NGS local assembly | North China | Northern_WildBoar |
| SChineseWB10 | NGS local assembly | South China | Southern_WildBoar |
| SChineseWB13 | NGS local assembly | South China | Southern_WildBoar |
| SChineseWB103 | NGS local assembly | South China | Southern_WildBoar |
| SChineseWB205 | NGS local assembly | South China | Southern_WildBoar |
| SChineseWB01 | NGS local assembly | South China | Southern_WildBoar |
| SChineseWB02 | NGS local assembly | South China | Southern_WildBoar |
| SChineseWB29U12 | NGS local assembly | South China | Southern_WildBoar |
| SChineseWB29U14 | NGS local assembly | South China | Southern_WildBoar |
| SChineseWB29U16 | NGS local assembly | South China | Southern_WildBoar |
| SChineseWB10 | NGS local assembly | South China | Southern_WildBoar |
| DQ409327 | Downloaded | Phacochoerus africanus | warthog |

**Table S2: Model selection and likelihood-ratio test of concatenated sequence of mitochondrial genes**

| Model | Categories | lnL | #p | AIC | BIC | 2ΔlnL | p value (LRT) |
| --- | --- | --- | --- | --- | --- | --- | --- |
| 1 ω model |  | -15150.01818 | 48 | 30396.03635 | 15343.72649 |  |  |
| 2 ω model | 1.Internal(I)/Terminal(T) | -15138.90892 | 49 | 30375.81785 | 15336.65283 | 22.218504 | 2.46E-06 |
|  | **2.Siberian/Vietnam** | **-15093.4356** | **49** | **30284.87119** | **15291.1795** | **113.165158** | **0.00E+00** |
|  | 3.Domestic(D)/Wild(W) | -15116.82134 | 49 | 30331.64267 | 15314.56524 | 66.393682 | 4.44E-16 |
| 3 ω model | 1.I/SiberianT/VietnamT | -15093.17516 | 50 | 30286.35032 | 15294.95465 | 91.467528 | 0.00E+00 |
|  | 2.Siberian/VietnamW/VietnamD | -15222.12958 | 50 | 30544.25915 | 15423.90907 | 257.387958 | 0.00E+00 |
|  |  |  |  |  |  | 144.2228 | 0.00E+00 |
| 4 ω model | 1.I/SiberianT/VietnamDT/VietnamWT | -15125.16811 | 51 | 30352.33621 | 15330.98319 | 63.985894 | 1.22E-15 |
| free ω model | -15084.25432 | 93 | 30354.50865 | 15459.56418 | 18.362546 | 1.00E+00 |  |

**Table S3: Model selection and likelihood-ratio test of ND1**

| Model | Categories | lnL | | #p | AIC | BIC | 2ΔAIC | | p value (LRT) | | | | |  |  |
| --- | --- | --- | --- | --- | --- | --- | --- | --- | --- | --- | --- | --- | --- | --- | --- |
| 1 ω model |  | -1208.15692 | | 48 | 2512.31384 | 1401.865231 |  | |  | |  | | | |  |
| 2 ω model | 1.Internal(I)/Terminal(T) | -1208.15692 | 49 | | 2514.31384 | 1405.900821 | | 4 | |  | | vs.1ω | | | |
|  | 2.Siberian/Vietnam | -1208.181936 | | 49 | 2514.363872 | 1405.925837 | 4.100064 | |  | | vs.1ω | | | |  |
|  | 3.Domestic(D)/Wild(W) | -1208.15692 | | 49 | 2514.31384 | 1405.900821 | 4 | |  | | vs.1ω | | | |  |
| 3 ω model | 1.Internal(I)/SiberianT/VietnamT | -1208.15692 | | 50 | 2516.31384 | 1409.936411 | 4 | |  | | vs.2ω1 | | | |  |
|  | 2.Siberian/VietnamW/VietnamD | -1208.157114 | | 50 | 2516.314228 | 1409.936605 | 3.900712 | |  | | vs.2ω2 | | | |  |
|  |  |  | |  |  |  |  | |  | | vs.1ω | | | |  |
| 4 ω model | 1.I/SiberianT/VietnamDT/VietnamWT | -1208.15692 | | 51 | 2518.31384 | 1413.972001 | 0 | |  | |  | | | |  |
| free ω model | | -1208.154395 | | 93 | 2602.30879 | 1583.464248 | 179.9899 | |  | | vs.1ω | | | |  |
| note:only p values <0.05 are listed | | | | | | | | | | | | |  |  |  |

**Table S4: Model selection and likelihood-ratio test of ND2**

| Model | Categories | lnL | #p | AIC | BIC | 2ΔAIC | p value (LRT) | |
| --- | --- | --- | --- | --- | --- | --- | --- | --- |
| 1 ω model |  | -1413.69 | 48 | 2923.388574 | 1607.402598 |  |  |  |
| 2 ω model | 1.Internal(I)/Terminal(T) | -1413.42 | 49 | 2924.849278 | 1611.16854 | 2.921408 |  | vs.1ω |
|  | 2.Siberian/Vietnam | -1413.66 | 49 | 2925.317742 | 1611.402772 | 3.858336 |  | vs.1ω |
|  | 3.Domestic(D)/Wild(W) | -1412.86 | 49 | 2923.727684 | 1610.607743 | 0.67822 |  | vs.1ω |
| 3 ω model | 1.Internal(I)/SiberianT/VietnamT | -1413.42 | 50 | 2926.848186 | 1615.203584 | 3.997816 |  | vs.2ω1 |
|  | 2.Siberian/VietnamW/VietnamD | -1412.80 | 50 | 2925.593752 | 1614.576367 | 0.55202 |  | vs.2ω2 |
|  |  |  |  |  |  |  |  | vs.1ω |
| 4 ω model | 1.I/SiberianT/VietnamDT/VietnamWT | -1412.863368 | 51 | 2927.726736 | 1618.678449 | 1.12145 |  |  |
| free ω model | | -1403.44 | 93 | 2992.886564 | 1778.753135 | 138.99598 |  | vs.1ω |
| note:only p values <0.05 are listed | | | | | | | | |

**Table S5: Model selection and likelihood-ratio test of COX1**

| Model | Categories | lnL | #p | AIC | BIC | 2ΔAIC | p value (LRT) | |
| --- | --- | --- | --- | --- | --- | --- | --- | --- |
| 1 ω model |  | -2096.16 | 48 | 4288.32019 | 2289.868406 |  |  |  |
| 2 ω model | 1.Internal(I)/Terminal(T) | -2096.16 | 49 | 4290.319206 | 2293.903504 | 3.998032 |  | vs.1ω |
|  | 2.Siberian/Vietnam | -2096.16 | 49 | 4290.319206 | 2293.903504 | 3.998032 |  | vs.1ω |
|  | 3.Domestic(D)/Wild(W) | -2096.16 | 49 | 4290.319206 | 2293.903504 | 3.998032 |  | vs.1ω |
| 3 ω model | 1.Internal(I)/SiberianT/VietnamT | -2096.16 | 50 | 4292.319206 | 2297.939094 | 4 |  | vs.2ω1 |
|  | 2.Siberian/VietnamW/VietnamD | -2096.16 | 50 | 4292.319206 | 2297.939094 | 4 |  | vs.2ω2 |
|  |  |  |  |  |  |  |  | vs.1ω |
| 4 ω model | 1.I/SiberianT/VietnamDT/VietnamWT | -2096.159603 | 51 | 4294.319206 | 2301.974684 | 0 |  |  |
| free ω model | | -2096.16 | 93 | 4378.320356 | 2471.470031 | 180.000332 |  | vs.1ω |
| note:only p values <0.05 are listed | | | | | | | | |

**Table S6: Model selection and likelihood-ratio test of COX2**

| Model | Categories | | lnL | | #p | | AIC | | BIC | | 2ΔAIC | | p value (LRT) | | | |
| --- | --- | --- | --- | --- | --- | --- | --- | --- | --- | --- | --- | --- | --- | --- | --- | --- |
| 1 ω model |  | | -890.55 | | 48 | | 1877.097642 | | 1084.257132 | |  | |  | |  | |
| 2 ω model | 1.Internal(I)/Terminal(T) | | -890.55 | | 49 | | 1879.097254 | | 1088.292528 | | 3.999224 | |  | | vs.1ω | |
|  | 2.Siberian/Vietnam | | -890.55 | | 49 | | 1879.097414 | | 1088.292608 | | 3.999544 | |  | | vs.1ω | |
|  | 3.Domestic(D)/Wild(W) | | -890.55 | | 49 | | 1879.097482 | | 1088.292642 | | 3.99968 | |  | | vs.1ω | |
| 3 ω model | 1.Internal(I)/SiberianT/VietnamT | | -890.55 | | 50 | | 1881.097518 | | 1092.32825 | | 4.000528 | |  | | vs.2ω1 | |
|  | 2.Siberian/VietnamW/VietnamD | | -890.55 | | 50 | | 1881.097412 | | 1092.328197 | | 3.999996 | |  | | vs.2ω2 | |
|  |  | |  | |  | |  | |  | |  | |  | | vs.1ω | |
| 4 ω model | 1.I/SiberianT/VietnamDT/VietnamWT | | -890.548555 | | 51 | | 1883.09711 | | 1096.363636 | | 0.000408 | |  | |  | |
| free ω model | | -890.55 | | 93 | | 1967.096904 | | 1265.858305 | | 179.998524 | |  | | vs.1ω | |  |
| note:only p values <0.05 are listed | | | | | | | | | | | | | | | |  |

**Table S7: Model selection and likelihood-ratio test of ATP8**

| Model | Categories | lnL | #p | AIC | BIC | p value (LRT) |  |
| --- | --- | --- | --- | --- | --- | --- | --- |
| 1 ω model |  | -254.175902 | 48 | -252.269644 | 15023.82122 |  |  |
| 2 ω model | 1.Internal(I)/Terminal(T) | -252.774669 | 49 | -252.269644 | 14990.63748 |  | vs.1ω |
|  | 2.Siberian/Vietnam | -252.774665 | 49 | -252.269644 | 14990.25463 |  | vs.1ω |
|  | 3.Domestic(D)/Wild(W) | -252.774617 | 49 | -252.269644 | 15006.80989 |  | vs.1ω |
| 3 ω model | 1.Internal(I)/SiberianT/VietnamT | -252.774633 | 50 | -252.269644 | 14994.80461 |  | vs.2ω1 |
|  | 2.Siberian/VietnamW/VietnamD | -252.774665 | 50 | -252.269644 | 14993.059 |  | vs.2ω2 |
|  |  |  |  |  |  |  | vs.1ω |
| 4 ω model | 1.I/SiberianT/VietnamDT/VietnamWT | -252.774615 | 51 | -129.5461074 | 27804.95312 |  |  |
| free ω model | -252.774641 | 93 | -252.269954 | 15168.11007 |  | vs.1ω |  |

Table S8: Model selection and likelihood-ratio test of ATP6

| Model | Categories | | lnL | | #p | | AIC | | BIC | | 2ΔAIC | | p value (LRT) | | | |  |
| --- | --- | --- | --- | --- | --- | --- | --- | --- | --- | --- | --- | --- | --- | --- | --- | --- | --- |
| 1 ω model | |  | | -891.66 | | 48 | | 1879.312974 | | 1085.364798 | |  | |  |  | | |
| 2 ω model | | 1.Internal(I)/Terminal(T) | | -891.56 | | 49 | | 1881.116538 | | 1089.30217 | | 3.607128 | |  | vs.1ω | | |
|  | 2.Siberian/Vietnam | | -891.63 | | 49 | | 1881.25303 | | 1089.370416 | | 3.880112 | |  | | | vs.1ω |  |
|  | 3.Domestic(D)/Wild(W) | | -891.04 | | 49 | | 1880.071286 | | 1088.779544 | | 1.516624 | |  | | | vs.1ω |  |
| 3 ω model | | 1.Internal(I)/SiberianT/VietnamT | | -890.69 | | 50 | | 1881.379356 | | 1092.469169 | | 0.525636 | |  | vs.2ω1 | | |
|  | 2.Siberian/VietnamW/VietnamD | | -891.04 | | 50 | | 1882.071378 | | 1092.81518 | | 1.636696 | |  | | | vs.2ω2 |  |
|  |  | |  | |  | |  | |  | |  | |  | | | vs.1ω |  |
| 4 ω model | | 1.I/SiberianT/VietnamDT/VietnamWT | | -890.168556 | | 51 | | 1882.337112 | | 1095.983637 | | 1.042244 | |  |  | | |
| free ω model | | | -886.89 | | 93 | | 1959.77135 | | 1262.195528 | | 160.916752 | |  | | | vs.1ω |  |
| note:only p values <0.05 are listed | | | | | | | | | | | | | | | | |  |

Table S9: Model selection and likelihood-ratio test of COX3

| Model | Categories | lnL | #p | AIC | BIC | 2ΔAIC | p value (LRT) | |
| --- | --- | --- | --- | --- | --- | --- | --- | --- |
| 1 ω model |  | -1024.32 | 48 | 2144.64976 | 1218.033191 |  |  |  |
| 2 ω model | 1.Internal(I)/Terminal(T) | -1023.80 | 49 | 2145.606024 | 1221.546913 | 1.912528 |  | vs.1ω |
|  | 2.Siberian/Vietnam | -1022.41 | 49 | 2142.82937 | 1220.158586 | -3.64078 |  | vs.1ω |
|  | 3.Domestic(D)/Wild(W) | -1024.32 | 49 | 2146.64974 | 1222.068771 | 3.99996 |  | vs.1ω |
| 3 ω model | 1.Internal(I)/SiberianT/VietnamT | -1022.41 | 50 | 2144.829328 | 1224.194155 | -1.553392 |  | vs.2ω1 |
|  | 2.Siberian/VietnamW/VietnamD | -1022.41 | 50 | 2144.829372 | 1224.194177 | 4.000004 |  | vs.2ω2 |
|  |  |  |  |  |  |  |  | vs.1ω |
| 4 ω model | 1.I/SiberianT/VietnamDT/VietnamWT | -1022.414665 | 51 | 2146.82933 | 1228.229746 | 2E-06 |  |  |
| free ω model | | -1022.41 | 93 | 2230.829392 | 1397.724549 | 172.359264 |  | vs.1ω |
| note:only p values <0.05 are listed | | | | | | | | |

Table S10: Model selection and likelihood-ratio test of ND3

| Model | Categories | lnL | | #p | | AIC | | BIC | | 2ΔAIC | | p value (LRT) | | | |  |
| --- | --- | --- | --- | --- | --- | --- | --- | --- | --- | --- | --- | --- | --- | --- | --- | --- |
| 1 ω model |  | -432.37 | | 48 | | 960.735062 | | 626.0758422 | |  | |  | |  | |  |
| 2 ω model | 1.Internal(I)/Terminal(T) | -430.98 | | 49 | | 959.967752 | | 628.727777 | | -1.53462 | |  | | vs.1ω | |  |
|  | 2.Siberian/Vietnam | -432.37 | | 49 | | 962.735004 | | 630.111403 | | 3.999884 | |  | | vs.1ω | |  |
|  | 3.Domestic(D)/Wild(W) | -430.98 | | 49 | | 959.967894 | | 628.727848 | | -1.534336 | |  | | vs.1ω | |  |
| 3 ω model | 1.Internal(I)/SiberianT/VietnamT | -430.98 | | 50 | | 961.967692 | | 632.7633369 | | 3.99988 | |  | | vs.2ω1 | |  |
|  | 2.Siberian/VietnamW/VietnamD | -432.37 | | 50 | | 964.734988 | | 634.1469849 | | 3.999968 | |  | | vs.2ω2 | |  |
|  |  |  | |  | |  | |  | |  | |  | | vs.1ω | |  |
| 4 ω model | 1.I/SiberianT/VietnamDT/VietnamWT | -430.983826 | | 51 | | 963.967652 | | 636.7989067 | | 4E-05 | |  | |  | |  |
| free ω model | | | -430.98 | | 93 | | 1047.967582 | | 806.293644 | | 174.46504 | |  | | vs.1ω | |
| note:only p values <0.05 are listed | | | | | | | | | | | | | | | | |

Table S11: Model selection and likelihood-ratio test of ND4L

| Model | Categories | lnL | | #p | | AIC | | BIC | | 2ΔAIC | | p value (LRT) | | | |  |
| --- | --- | --- | --- | --- | --- | --- | --- | --- | --- | --- | --- | --- | --- | --- | --- | --- |
| 1 ω model |  | -392.99 | | 48 | | 881.980488 | | 586.6985552 | |  | |  | |  | |  |
| 2 ω model | 1.Internal(I)/Terminal(T) | -392.65 | | 49 | | 883.298934 | | 590.393368 | | 2.636892 | |  | | vs.1ω | |  |
|  | 2.Siberian/Vietnam | -392.65 | | 49 | | 883.300736 | | 590.394269 | | 2.640496 | |  | | vs.1ω | |  |
|  | 3.Domestic(D)/Wild(W) | -392.99 | | 49 | | 883.97936 | | 590.733581 | | 3.997744 | |  | | vs.1ω | |  |
| 3 ω model | 1.Internal(I)/SiberianT/VietnamT | -392.65 | | 50 | | 885.297674 | | 594.4283279 | | 3.99748 | |  | | vs.2ω1 | |  |
|  | 2.Siberian/VietnamW/VietnamD | -393.22 | | 50 | | 886.44614 | | 595.0025609 | | 6.290808 | |  | | vs.2ω2 | |  |
|  |  |  | |  | |  | |  | |  | |  | | vs.1ω | |  |
| 4 ω model | 1.I/SiberianT/VietnamDT/VietnamWT | -390.732681 | | 51 | | 883.465362 | | 596.5477617 | | 3.832312 | |  | |  | |  |
| free ω model | | | -391.40 | | 93 | | 968.80851 | | 766.714108 | | 173.656044 | |  | | vs.1ω | |
| note:only p values <0.05 are listed | | | | | | | | | | | | | | | | |

Table S12: Model selection and likelihood-ratio test of ND4

| Model | Categories | lnL | #p | AIC | BIC | 2ΔAIC | p value (LRT) | |
| --- | --- | --- | --- | --- | --- | --- | --- | --- |
| 1 ω model |  | -1730.90 | 48 | 3557.79441 | 1924.605516 |  |  |  |
| 2 ω model | 1.Internal(I)/Terminal(T) | -1730.90 | 49 | 3559.79441 | 1928.641106 | 4 |  | vs.1ω |
|  | 2.Siberian/Vietnam | -1730.90 | 49 | 3559.790342 | 1928.639072 | 3.991864 |  | vs.1ω |
|  | 3.Domestic(D)/Wild(W) | -1730.90 | 49 | 3559.791734 | 1928.639768 | 3.994648 |  | vs.1ω |
| 3 ω model | 1.Internal(I)/SiberianT/VietnamT | -1730.90 | 50 | 3561.792378 | 1932.67568 | 3.995936 |  | vs.2ω1 |
|  | 2.Siberian/VietnamW/VietnamD | -1730.89 | 50 | 3561.788304 | 1932.673643 | 3.995924 |  | vs.2ω2 |
|  |  |  |  |  |  |  |  | vs.1ω |
| 4 ω model | 1.I/SiberianT/VietnamDT/VietnamWT | -1730.894917 | 51 | 3563.789834 | 1936.709998 | 0.002544 |  |  |
| free ω model | | -1730.89 | 93 | 3647.782284 | 2106.200995 | 179.975748 |  | vs.1ω |
| note:only p values <0.05 are listed | | | | | | | | |

Table S13: Model selection and likelihood-ratio test of ND5

| Model | Categories | lnL | | #p | | AIC | | BIC | | 2ΔAIC | | p value (LRT) | | | |  |
| --- | --- | --- | --- | --- | --- | --- | --- | --- | --- | --- | --- | --- | --- | --- | --- | --- |
| 1 ω model |  | -2326.88 | | 48 | | 4749.756504 | | 2520.586563 | |  | |  | |  | |  |
| 2 ω model | 1.Internal(I)/Terminal(T) | -2326.63 | | 49 | | 4751.253492 | | 2524.370647 | | 2.993976 | |  | | vs.1ω | |  |
|  | 2.Siberian/Vietnam | -2326.07 | | 49 | | 4750.148756 | | 2523.818279 | | 0.784504 | |  | | vs.1ω | |  |
|  | 3.Domestic(D)/Wild(W) | -2325.77 | | 49 | | 4749.545894 | | 2523.516848 | | -0.42122 | |  | | vs.1ω | |  |
| 3 ω model | 1.Internal(I)/SiberianT/VietnamT | -2326.63 | | 50 | | 4753.25238 | | 2528.405681 | | 3.997776 | |  | | vs.2ω1 | |  |
|  | 2.Siberian/VietnamW/VietnamD | -2324.45 | | 50 | | 4748.891522 | | 2526.225252 | | -2.514468 | |  | | vs.2ω2 | |  |
|  |  |  | |  | |  | |  | |  | |  | | vs.1ω | |  |
| 4 ω model | 1.I/SiberianT/VietnamDT/VietnamWT | -2325.760898 | | 51 | | 4753.521796 | | 2531.575979 | | 1.730584 | |  | |  | |  |
| free ω model | | | -2319.66 | | 93 | | 4825.329638 | | 2694.974672 | | 151.146268 | |  | | vs.1ω | |
| note:only p values <0.05 are listed | | | | | | | | | | | | | | | | |

Table S14: Model selection and likelihood-ratio test of Cytb

| Model | Categories | lnL | | #p | | AIC | | BIC | | 2ΔAIC | | p value (LRT) | | | |  |
| --- | --- | --- | --- | --- | --- | --- | --- | --- | --- | --- | --- | --- | --- | --- | --- | --- |
| 1 ω model |  | -1983.97 | | 48 | | 4063.935836 | | 2107.7426 | |  | |  | |  | |  |
| 2 ω model | 1.Internal(I)/Terminal(T) | -1983.95 | | 49 | | 4065.897064 | | 2110.301853 | | 3.922456 | |  | | vs.1ω | |  |
|  | 2.Siberian/Vietnam | -1981.86 | | 49 | | **4061.716482** | | 2108.211562 | | -4.438708 | | 0.04 | | vs.1ω | |  |
|  | 3.Domestic(D)/Wild(W) | -1982.74 | | 49 | | 4063.483222 | | 2109.094932 | | -0.905228 | |  | | vs.1ω | |  |
| 3 ω model | 1.Internal(I)/SiberianT/VietnamT | -1984.61 | | 50 | | 4069.218518 | | 2113.541219 | | 6.642908 | |  | | vs.2ω1 | |  |
|  | 2.Siberian/VietnamW/VietnamD | -1982.68 | | 50 | | 4065.362768 | | 2111.613344 | | 7.292572 | |  | | vs.2ω2 | |  |
|  |  |  | |  | |  | |  | |  | |  | | vs.1ω | |  |
| 4 ω model | 1.I/SiberianT/VietnamDT/VietnamWT | -1979.918385 | | 51 | | 4061.83677 | | 2185.733466 | | 9.381748 | |  | |  | |  |
| free ω model | | | -1970.98 | | 93 | | 4127.96202 | | 2255.166733 | | 128.052368 | |  | | vs.1ω | |
| note:only p values <0.05 are listed | | | | | | | | | | | | | | | | |

Table S15: Cytb sequences of wild boars from broader southern and northern regions used to evaluate Ka/Ks.

| Group | Genebank ID | Sample name | Location |
| --- | --- | --- | --- |
| Southern_Wildboar | GU135837.1 | ZhejiangWB | Zhejiang,China |
| Southern_Wildboar | GU135836.1 | ZhejiangWB | Zhejiang,China |
| Southern_Wildboar | GU135835.1 | ZhejiangWB | Zhejiang,China |
| Southern_Wildboar | GU135834.1 | ZhejiangWB | Zhejiang,China |
| Southern_Wildboar | GU135831.1 | ZhejiangWB | Zhejiang,China |
| Southern_Wildboar | GU135830.1 | ZhejiangWB | Zhejiang,China |
| Southern_Wildboar | GU135829.1 | ZhejiangWB | Zhejiang,China |
| Southern_Wildboar | GU135828.1 | ZhejiangWB | Zhejiang,China |
| Southern_Wildboar | GU135787.1 | ZhejiangWB | Zhejiang,China |
| Southern_Wildboar | GU135786.1 | ZhejiangWB | Zhejiang,China |
| Southern_Wildboar | GU135826.1 | LinanWB | Zhejiang,China |
| Southern_Wildboar | GU135806.1 | XiangshanWB | Zhejiang,China |
| Southern_Wildboar | GU135805.1 | TaizhouWB | Zhejiang,China |
| Southern_Wildboar | GU135822.1 | JiangshanWB | Zhejiang,China |
| Southern_Wildboar | GU135821.1 | JiangshanWB | Zhejiang,China |
| Southern_Wildboar | EF545579.1 | JiangxiWB | Jiangxi,China |
| Southern_Wildboar | GU135790.1 | JiangxiWB | Jiangxi,China |
| Southern_Wildboar | GU135825.1 | JiangxiWB | Jiangxi,China |
| Southern_Wildboar | GU135824.1 | JiangxiWB | Jiangxi,China |
| Southern_Wildboar | GU135823.1 | JiangxiWB | Jiangxi,China |
| Southern_Wildboar | EF545572.1 | HainanWB | Hainan,China |
| Southern_Wildboar | KP681245.1 | HainanWB | Hainan,China |
| Southern_Wildboar | GU135820.1 | HainanWB | Hainan,China |
| Southern_Wildboar | GU135819.1 | HainanWB | Hainan,China |
| Southern_Wildboar | GU135818.1 | HainanWB | Hainan,China |
| Southern_Wildboar | GU135817.1 | HainanWB | Hainan,China |
| Southern_Wildboar | GU135816.1 | HainanWB | Hainan,China |
| Southern_Wildboar | GU135815.1 | HainanWB | Hainan,China |
| Southern_Wildboar | GU135814.1 | HainanWB | Hainan,China |
| Southern_Wildboar | GU135813.1 | HainanWB | Hainan,China |
| Southern_Wildboar | GU135812.1 | HainanWB | Hainan,China |
| Southern_Wildboar | GU135811.1 | HainanWB | Hainan,China |
| Southern_Wildboar | GU135810.1 | HainanWB | Hainan,China |
| Southern_Wildboar | GU135809.1 | HainanWB | Hainan,China |
| Southern_Wildboar | GU135789.1 | HainanWB | Hainan,China |
| Southern_Wildboar | GU135788.1 | HainanWB | Hainan,China |
| Southern_Wildboar | DQ315598.1 | HainanWB | Hainan,China |
| Southern_Wildboar | DQ315597.1 | HainanWB | Hainan,China |
| Southern_Wildboar | EF545568.1 | YunanaWB | Yunnan,China |
| Southern_Wildboar | DQ315600.1 | YunnanWB | Yunnan,China |
| Southern_Wildboar | DQ315599.1 | YunnanWB | Yunnan,China |
| Southern_Wildboar | EF545586.1 | YunnanWB | Yunnan,China |
| Southern_Wildboar | EF545573.1 | YunnanWB | Yunnan,China |
| Southern_Wildboar | EF545570.1 | FujianWB | Fujian,China |
| Southern_Wildboar | EF545569.1 | FujianWB | Fujian,China |
| Southern_Wildboar | KX982644.1 | VietnamWB | Vietnam |
| Southern_Wildboar | KX982642.1 | VietnamWB | Vietnam |
| Southern_Wildboar | KX982641.1 | VietnamWB | Vietnam |
| Southern_Wildboar | KX982640.1 | VietnamWB | Vietnam |
| Southern_Wildboar | KX982639.1 | VietnamWB | Vietnam |
| Southern_Wildboar | KX982638.1 | VietnamWB | Vietnam |
| Southern_Wildboar | KX982637.1 | VietnamWB | Vietnam |
| Southern_Wildboar | KX982636.1 | VietnamWB | Vietnam |
| Southern_Wildboar | DQ315603.1 | VietnamWB | Vietnam |
| Southern_Wildboar | EF545584.1 | VietnamWB | Vietnam |
| Southern_Wildboar | DQ444704.1 | LaosWB | Laos |
| Southern_Wildboar | Assembly | SCWB10 | South China |
| Southern_Wildboar | Assembly | SCWB13 | South China |
| Southern_Wildboar | Assembly | SCWB103 | South China |
| Southern_Wildboar | Assembly | SCWB205 | South China |
| Southern_Wildboar | Assembly | SCWB01 | South China |
| Southern_Wildboar | Assembly | SCWB02 | South China |
| Southern_Wildboar | Assembly | SCWB29U14 | South China |
| Southern_Wildboar | Assembly | SCWB29U16 | South China |
| Northern_Wildboar | Assembly | KWB1 | Korea |
| Northern_Wildboar | Assembly | KWB10 | Korea |
| Northern_Wildboar | Assembly | KWB2 | Korea |
| Northern_Wildboar | Assembly | KWB3 | Korea |
| Northern_Wildboar | Assembly | KWB4 | Korea |
| Northern_Wildboar | Assembly | KWB5 | Korea |
| Northern_Wildboar | Assembly | KWB6 | Korea |
| Northern_Wildboar | Assembly | KWB7 | Korea |
| Northern_Wildboar | Assembly | KWB8 | Korea |
| Northern_Wildboar | Assembly | KWB9 | Korea |
| Northern_Wildboar | Assembly | NECWB1 | North China |
| Northern_Wildboar | Assembly | NECWB3 | North China |
| Northern_Wildboar | Assembly | NECWB4 | North China |
| Northern_Wildboar | Assembly | NCWB30U08 | North China |
| Northern_Wildboar | HM010474.1 | RussiaWB | Russia |
| Northern_Wildboar | HM010473.1 | RussiaWB | Russia |
| Northern_Wildboar | HM010471.1 | RussiaWB | Russia |
| Northern_Wildboar | HM010470.1 | RussiaWB | Russia |
| Northern_Wildboar | HM010469.1 | RussiaWB | Russia |
| Northern_Wildboar | HM010468.1 | RussiaWB | Russia |
| Northern_Wildboar | HM010467.1 | RussiaWB | Russia |
| Northern_Wildboar | HM010466.1 | RussiaWB | Russia |
| Northern_Wildboar | HM010465.1 | RussiaWB | Russia |
| Northern_Wildboar | HM010464.1 | RussiaWB | Russia |
| Northern_Wildboar | HM010463.1 | RussiaWB | Russia |
| Northern_Wildboar | HM010462.1 | RussiaWB | Russia |
| Northern_Wildboar | HM010461.1 | RussiaWB | Russia |
| Northern_Wildboar | KM215177.1 | MongliaWB | Monglia |
| Northern_Wildboar | KM215174.1 | MongliaWB | Monglia |
| Northern_Wildboar | KM215173.1 | MongliaWB | Monglia |
| Northern_Wildboar | KM215170.1 | MongliaWB | Monglia |
| Northern_Wildboar | KM215168.1 | MongliaWB | Monglia |
| Northern_Wildboar | KM215165.1 | MongliaWB | Monglia |
| Northern_Wildboar | AY830171.1 | KoreaWB | Korea |
| Northern_Wildboar | AY830167.1 | KoreaWB | Korea |
| Northern_Wildboar | AY830165.1 | KoreaWB | Korea |
| Northern_Wildboar | AY830162.1 | KoreaWB | Korea |
| Northern_Wildboar | AY830159.1 | KoreaWB | Korea |
| Northern_Wildboar | AY634187.1 | KoreaWB | Korea |
| Northern_Wildboar | AY634184.1 | KoreaWB | Korea |
| Northern_Wildboar | EU090702.1 | KoreaWB | Korea |
| Northern_Wildboar | DQ268530.1 | KoreaWB | Korea |
| Northern_Wildboar | DQ207753.1 | KoreaWB | Korea |
| Northern_Wildboar | DQ207754.1 | KoreaWB | Korea |
| Northern_Wildboar | DQ207755.1 | KoreaWB | Korea |
| Northern_Wildboar | GU135808.1 | NortheastWB | North China |
| Northern_Wildboar | GU135807.1 | NortheastWB | North China |
| Northern_Wildboar | GU135794.1 | NortheastWB | North China |
| Northern_Wildboar | GU135793.1 | NortheastWB | North China |
| Northern_Wildboar | GU135792.1 | NortheastWB | North China |
| Northern_Wildboar | GU135791.1 | NortheastWB | North China |
| Northern_Wildboar | EF545580.1 | NortheastWB | North China |
| Northern_Wildboar | EU333163.1 | NortheastWB | North China |
| Northern_Wildboar | KP765605.1 | ChangbaishanWB | North China |
| Northern_Wildboar | This study | RussiaWildBoarCO | Russia |
| Northern_Wildboar | This study | RussiaWildBoarT1 | Russia |
| Northern_Wildboar | This study | RussiaWildBoarT2 | Russia |
| Northern_Wildboar | This study | RussiaWildBoarT3 | Russia |
| Northern_Wildboar | This study | RussiaWildBoarW1 | Russia |
| Northern_Wildboar | This study | RussiaWildBoarW2 | Russia |
| Northern_Wildboar | This study | RussiaWildBoarZK | Russia |

Table S16: Sequences used to conduct BSP analysis.

| GeneBank ID | Location | Group | Note |
| --- | --- | --- | --- |
| EF533693.1 | Korea | Northern_WildBoar | sanger |
| EF533690.1 | Korea | Northern_WildBoar | sanger |
| EF533689.1 | Korea | Northern_WildBoar | sanger |
| EF533688.1 | Korea | Northern_WildBoar | sanger |
| EF533687.1 | Korea | Northern_WildBoar | sanger |
| EF533686.1 | Korea | Northern_WildBoar | sanger |
| EF533685.1 | Korea | Northern_WildBoar | sanger |
| AY879784.1 | Korea | Northern_WildBoar | sanger |
| AY879783.1 | Korea | Northern_WildBoar | sanger |
| AY879782.1 | Korea | Northern_WildBoar | sanger |
| AY879780.1 | Korea | Northern_WildBoar | sanger |
| AY879779.1 | Korea | Northern_WildBoar | sanger |
| AY879778.1 | Korea | Northern_WildBoar | sanger |
| AY879777.1 | Korea | Northern_WildBoar | sanger |
| AY879775.1 | Korea | Northern_WildBoar | sanger |
| AY879772.1 | Korea | Northern_WildBoar | sanger |
| AY879771.1 | Korea | Northern_WildBoar | sanger |
| AY534288.1 | Korea | Northern_WildBoar | sanger |
| AY534287.1 | Korea | Northern_WildBoar | sanger |
| AY534284.1 | Korea | Northern_WildBoar | sanger |
| AY534282.1 | Korea | Northern_WildBoar | sanger |
| AY534285.1 | Korea | Northern_WildBoar | sanger |
| AY534283.1 | Korea | Northern_WildBoar | sanger |
| AY429458.1 | Korea | Northern_WildBoar | sanger |
| AY429457.1 | Korea | Northern_WildBoar | sanger |
| KP027465.1 | Mongolia | Northern_WildBoar | sanger |
| KP027462.1 | Mongolia | Northern_WildBoar | sanger |
| KP027460.1 | Mongolia | Northern_WildBoar | sanger |
| KP027459.1 | Mongolia | Northern_WildBoar | sanger |
| KP027458.1 | Mongolia | Northern_WildBoar | sanger |
| KP027452.1 | Mongolia | Northern_WildBoar | sanger |
| KP027450.1 | Mongolia | Northern_WildBoar | sanger |
| KP027449.1 | Mongolia | Northern_WildBoar | sanger |
| KP027448.1 | Mongolia | Northern_WildBoar | sanger |
| HM026639.1 | Northeast_China | Northern_WildBoar | sanger |
| HM010488.1 | Russia | Northern_WildBoar | sanger |
| HM010487.1 | Russia | Northern_WildBoar | sanger |
| HM010486.1 | Russia | Northern_WildBoar | sanger |
| HM010485.1 | Russia | Northern_WildBoar | sanger |
| HM010484.1 | Russia | Northern_WildBoar | sanger |
| HM010483.1 | Russia | Northern_WildBoar | sanger |
| HM010482.1 | Russia | Northern_WildBoar | sanger |
| HM010481.1 | Russia | Northern_WildBoar | sanger |
| HM010480.1 | Russia | Northern_WildBoar | sanger |
| HM010479.1 | Russia | Northern_WildBoar | sanger |
| HM010478.1 | Russia | Northern_WildBoar | sanger |
| HM010477.1 | Russia | Northern_WildBoar | sanger |
| HM010476.1 | Russia | Northern_WildBoar | sanger |
| HM010475.1 | Russia | Northern_WildBoar | sanger |
| DQ379265.2 | Northeast_China | Northern_WildBoar | sanger |
| DQ379264.2 | Northeast_China | Northern_WildBoar | sanger |
| DQ379263.2 | Northeast_China | Northern_WildBoar | sanger |
| DQ379262.2 | Northeast_China | Northern_WildBoar | sanger |
| DQ496842.1 | Shaanxi_China | Northern_WildBoar | sanger |
| DQ496841.1 | Shaanxi_China | Northern_WildBoar | sanger |
| DQ496840.1 | Shaanxi_China | Northern_WildBoar | sanger |
| DQ496839.1 | Shaanxi_China | Northern_WildBoar | sanger |
| DQ496838.1 | Shaanxi_China | Northern_WildBoar | sanger |
| DQ496837.1 | Shaanxi_China | Northern_WildBoar | sanger |
| DQ496836.1 | Shaanxi_China | Northern_WildBoar | sanger |
| DQ496835.1 | Shaanxi_China | Northern_WildBoar | sanger |
| DQ496834.1 | Shaanxi_China | Northern_WildBoar | sanger |
| DQ496833.1 | Shaanxi_China | Northern_WildBoar | sanger |
| DQ496791.1 | Gansu_China | Northern_WildBoar | sanger |
| DQ496790.1 | Gansu_China | Northern_WildBoar | sanger |
| DQ496789.1 | Gansu_China | Northern_WildBoar | sanger |
| DQ496788.1 | Gansu_China | Northern_WildBoar | sanger |
| DQ496772.1 | Northeast_China | Northern_WildBoar | sanger |
| DQ496771.1 | Northeast_China | Northern_WildBoar | sanger |
| DQ496770.1 | Northeast_China | Northern_WildBoar | sanger |
| DQ496769.1 | Northeast_China | Northern_WildBoar | sanger |
| DQ496768.1 | Northeast_China | Northern_WildBoar | sanger |
| DQ496767.1 | Northeast_China | Northern_WildBoar | sanger |
| DQ496766.1 | Northeast_China | Northern_WildBoar | sanger |
| DQ496765.1 | Northeast_China | Northern_WildBoar | sanger |
| DQ496764.1 | Northeast_China | Northern_WildBoar | sanger |
| DQ496763.1 | Northeast_China | Northern_WildBoar | sanger |
| DQ496762.1 | Northeast_China | Northern_WildBoar | sanger |
| DQ496761.1 | Northeast_China | Northern_WildBoar | sanger |
| DQ496760.1 | Northeast_China | Northern_WildBoar | sanger |
| DQ496759.1 | Northeast_China | Northern_WildBoar | sanger |
| DQ496758.1 | Northeast_China | Northern_WildBoar | sanger |
| DQ496757.1 | Northeast_China | Northern_WildBoar | sanger |
| DQ496756.1 | Northeast_China | Northern_WildBoar | sanger |
| DQ496755.1 | Northeast_China | Northern_WildBoar | sanger |
| DQ496754.1 | Northeast_China | Northern_WildBoar | sanger |
| DQ496753.1 | Northeast_China | Northern_WildBoar | sanger |
| DQ496752.1 | Northeast_China | Northern_WildBoar | sanger |
| DQ496751.1 | Northeast_China | Northern_WildBoar | sanger |
| DQ496750.1 | Northeast_China | Northern_WildBoar | sanger |
| DQ496749.1 | Northeast_China | Northern_WildBoar | sanger |
| DQ496748.1 | Northeast_China | Northern_WildBoar | sanger |
| DQ496747.1 | Northeast_China | Northern_WildBoar | sanger |
| DQ496746.1 | Northeast_China | Northern_WildBoar | sanger |
| DQ496745.1 | Northeast_China | Northern_WildBoar | sanger |
| DQ496744.1 | Northeast_China | Northern_WildBoar | sanger |
| DQ207761.1 | Korea | Northern_WildBoar | sanger |
| DQ207760.1 | Korea | Northern_WildBoar | sanger |
| DQ207759.1 | Korea | Northern_WildBoar | sanger |
| DQ207758.1 | Korea | Northern_WildBoar | sanger |
| DQ207757.1 | Korea | Northern_WildBoar | sanger |
| DQ191228.1 | Korea | Northern_WildBoar | sanger |
| DQ191227.1 | Korea | Northern_WildBoar | sanger |
| DQ191225.1 | Korea | Northern_WildBoar | sanger |
| DQ191224.1 | Korea | Northern_WildBoar | sanger |
| DQ191223.1 | Korea | Northern_WildBoar | sanger |
| DQ191222.1 | Korea | Northern_WildBoar | sanger |
| DQ191221.1 | Korea | Northern_WildBoar | sanger |
| DQ191218.1 | Korea | Northern_WildBoar | sanger |
| DQ191217.1 | Korea | Northern_WildBoar | sanger |
| DQ191216.1 | Korea | Northern_WildBoar | sanger |
| DQ191215.1 | Korea | Northern_WildBoar | sanger |
| DQ191213.1 | Korea | Northern_WildBoar | sanger |
| DQ191212.1 | Korea | Northern_WildBoar | sanger |
| AY751460.1 | Northeast_China | Northern_WildBoar | sanger |
| AY574047.1 | Korean | Northern_WildBoar | sanger |
| KX982635.1 | Russia | Northern_WildBoar | sanger |
| KX982634.1 | Russia | Northern_WildBoar | sanger |
| KX982633.1 | Russia | Northern_WildBoar | sanger |
| KX982631.1 | Russia | Northern_WildBoar | sanger |
| KX982630.1 | Russia | Northern_WildBoar | sanger |
| KX982629.1 | Russia | Northern_WildBoar | sanger |
| EU090703.1 | Korea | Northern_WildBoar | sanger |
| EU090702.1 | Korea | Northern_WildBoar | sanger |
| DQ268530.1 | Korea | Northern_WildBoar | sanger |
| DQ207754.1 | Korea | Northern_WildBoar | sanger |
| EF545580.1 | Northeast_China | Northern_WildBoar | sanger |
| DQ207755.1 | Korea | Northern_WildBoar | sanger |
| KP765605.1 | Northeast_China | Northern_WildBoar | sanger |
| AY884642.1 | Gansu_China | Northern_WildBoar | sanger |
| FJ601526.1 | Northeast_China | Northern_WildBoar | sanger |
| FJ601527.1 | Northeast_China | Northern_WildBoar | sanger |
| FJ601528.1 | Northeast_China | Northern_WildBoar | sanger |
| FJ601529.1 | Northeast_China | Northern_WildBoar | sanger |
| AY884627.1 | Shanxi_China | Northern_WildBoar | sanger |
| AY884639.1 | Shanxi_China | Northern_WildBoar | sanger |
| AY884684.1 | Shanxi_China | Northern_WildBoar | sanger |
| DQ779422.1 | Korea | Northern_WildBoar | sanger |
| DQ779521.1 | Korea | Northern_WildBoar | sanger |
| DQ779522.1 | Korea | Northern_WildBoar | sanger |
| DQ779523.1 | Korea | Northern_WildBoar | sanger |
| DQ779524.1 | Korea | Northern_WildBoar | sanger |
| DQ779525.1 | Korea | Northern_WildBoar | sanger |
| DQ779526.1 | Korea | Northern_WildBoar | sanger |
| DQ779527.1 | Korea | Northern_WildBoar | sanger |
| NEChineseWB1 | North China | Northern_WildBoar | NGS local assembly |
| NEChineseWB3 | North China | Northern_WildBoar | NGS local assembly |
| NEChineseWB4 | North China | Northern_WildBoar | NGS local assembly |
| KoreanWB1 | North China | Northern_WildBoar | NGS local assembly |
| KoreanWB10 | North China | Northern_WildBoar | NGS local assembly |
| KoreanWB2 | North China | Northern_WildBoar | NGS local assembly |
| KoreanWB3 | North China | Northern_WildBoar | NGS local assembly |
| KoreanWB4 | North China | Northern_WildBoar | NGS local assembly |
| KoreanWB5 | North China | Northern_WildBoar | NGS local assembly |
| KoreanWB6 | North China | Northern_WildBoar | NGS local assembly |
| KoreanWB7 | North China | Northern_WildBoar | NGS local assembly |
| KoreanWB8 | North China | Northern_WildBoar | NGS local assembly |
| KoreanWB9 | North China | Northern_WildBoar | NGS local assembly |
| NChineseWB30U08 | North China | Northern_WildBoar | NGS local assembly |
| NChineseWB30U09 | North China | Northern_WildBoar | NGS local assembly |
| KP938215.1 | Vietnam | Southern_WildBoar | sanger |
| KP938214.1 | Vietnam | Southern_WildBoar | sanger |
| KP938213.1 | Vietnam | Southern_WildBoar | sanger |
| KP938212.1 | Vietnam | Southern_WildBoar | sanger |
| KP938211.1 | Vietnam | Southern_WildBoar | sanger |
| KP938210.1 | Vietnam | Southern_WildBoar | sanger |
| KP938209.1 | Vietnam | Southern_WildBoar | sanger |
| KP938208.1 | Vietnam | Southern_WildBoar | sanger |
| KP938207.1 | Vietnam | Southern_WildBoar | sanger |
| JQ898539.1 | Vietnam | Southern_WildBoar | sanger |
| JQ898538.1 | Vietnam | Southern_WildBoar | sanger |
| JQ898537.1 | Vietnam | Southern_WildBoar | sanger |
| JQ898536.1 | Vietnam | Southern_WildBoar | sanger |
| JQ898535.1 | Vietnam | Southern_WildBoar | sanger |
| JQ898532.1 | Vietnam | Southern_WildBoar | sanger |
| JQ898531.1 | Vietnam | Southern_WildBoar | sanger |
| AB326951.1 | Vietnam | Southern_WildBoar | sanger |
| AB326950.1 | Vietnam | Southern_WildBoar | sanger |
| AB326949.1 | Vietnam | Southern_WildBoar | sanger |
| AB326948.1 | Vietnam | Southern_WildBoar | sanger |
| AB326947.1 | Vietnam | Southern_WildBoar | sanger |
| AB326946.1 | Vietnam | Southern_WildBoar | sanger |
| AB326945.1 | Vietnam | Southern_WildBoar | sanger |
| AB326944.1 | Vietnam | Southern_WildBoar | sanger |
| AB326943.1 | Vietnam | Southern_WildBoar | sanger |
| AB326941.1 | Vietnam | Southern_WildBoar | sanger |
| AB326940.1 | Vietnam | Southern_WildBoar | sanger |
| AB326939.1 | Vietnam | Southern_WildBoar | sanger |
| AB326938.1 | Vietnam | Southern_WildBoar | sanger |
| AB326937.1 | Vietnam | Southern_WildBoar | sanger |
| AB326936.1 | Vietnam | Southern_WildBoar | sanger |
| AB326935.1 | Vietnam | Southern_WildBoar | sanger |
| AB326934.1 | Vietnam | Southern_WildBoar | sanger |
| AB326933.1 | Vietnam | Southern_WildBoar | sanger |
| AB252823.1 | Cambodia | Southern_WildBoar | sanger |
| AB302182.1 | Vietnam | Southern_WildBoar | sanger |
| AB306907.1 | Vietnam | Southern_WildBoar | sanger |
| AB306906.1 | Vietnam | Southern_WildBoar | sanger |
| AB306905.1 | Vietnam | Southern_WildBoar | sanger |
| AB306904.1 | Vietnam | Southern_WildBoar | sanger |
| AB306903.1 | Vietnam | Southern_WildBoar | sanger |
| AB306902.1 | Vietnam | Southern_WildBoar | sanger |
| AB306901.1 | Vietnam | Southern_WildBoar | sanger |
| AB306900.1 | Vietnam | Southern_WildBoar | sanger |
| JX068444.1 | Sichuan_China | Southern_WildBoar | sanger |
| JX068443.1 | Sichuan_China | Southern_WildBoar | sanger |
| JX068442.1 | Sichuan_China | Southern_WildBoar | sanger |
| JX068441.1 | Sichuan_China | Southern_WildBoar | sanger |
| JX068440.1 | Sichuan_China | Southern_WildBoar | sanger |
| AM779937.1 | Thailand | Southern_WildBoar | sanger |
| AM779936.1 | Thailand | Southern_WildBoar | sanger |
| AM779935.1 | Thailand | Southern_WildBoar | sanger |
| AM779934.1 | Thailand | Southern_WildBoar | sanger |
| AM779933.1 | Thailand | Southern_WildBoar | sanger |
| HM026640.1 | Zhejiang_China | Southern_WildBoar | sanger |
| EF590191.1 | Zhejiang_China | Southern_WildBoar | sanger |
| EF590185.1 | Zhejiang_China | Southern_WildBoar | sanger |
| EF590184.1 | Zhejiang_China | Southern_WildBoar | sanger |
| EF590180.1 | Zhejiang_China | Southern_WildBoar | sanger |
| EF590179.1 | Zhejiang_China | Southern_WildBoar | sanger |
| DQ379267.2 | Hainan_China | Southern_WildBoar | sanger |
| DQ379266.2 | Hainan_China | Southern_WildBoar | sanger |
| DQ496912.1 | Zhejiang_China | Southern_WildBoar | sanger |
| DQ496911.1 | Zhejiang_China | Southern_WildBoar | sanger |
| DQ496910.1 | Zhejiang_China | Southern_WildBoar | sanger |
| DQ496909.1 | Zhejiang_China | Southern_WildBoar | sanger |
| DQ496908.1 | Zhejiang_China | Southern_WildBoar | sanger |
| DQ496907.1 | Zhejiang_China | Southern_WildBoar | sanger |
| DQ496906.1 | Zhejiang_China | Southern_WildBoar | sanger |
| DQ496905.1 | Zhejiang_China | Southern_WildBoar | sanger |
| DQ496904.1 | Zhejiang_China | Southern_WildBoar | sanger |
| DQ496903.1 | Zhejiang_China | Southern_WildBoar | sanger |
| DQ496902.1 | Zhejiang_China | Southern_WildBoar | sanger |
| DQ496901.1 | Zhejiang_China | Southern_WildBoar | sanger |
| DQ496900.1 | Zhejiang_China | Southern_WildBoar | sanger |
| DQ496899.1 | Zhejiang_China | Southern_WildBoar | sanger |
| DQ496898.1 | Zhejiang_China | Southern_WildBoar | sanger |
| DQ496897.1 | Zhejiang_China | Southern_WildBoar | sanger |
| DQ496896.1 | Zhejiang_China | Southern_WildBoar | sanger |
| DQ496895.1 | Zhejiang_China | Southern_WildBoar | sanger |
| DQ496894.1 | Zhejiang_China | Southern_WildBoar | sanger |
| DQ496893.1 | Zhejiang_China | Southern_WildBoar | sanger |
| DQ496892.1 | Zhejiang_China | Southern_WildBoar | sanger |
| DQ496891.1 | Zhejiang_China | Southern_WildBoar | sanger |
| DQ496890.1 | Zhejiang_China | Southern_WildBoar | sanger |
| DQ496889.1 | Zhejiang_China | Southern_WildBoar | sanger |
| DQ496888.1 | Zhejiang_China | Southern_WildBoar | sanger |
| DQ496887.1 | Zhejiang_China | Southern_WildBoar | sanger |
| DQ496886.1 | Zhejiang_China | Southern_WildBoar | sanger |
| DQ496885.1 | Yunnan_China | Southern_WildBoar | sanger |
| DQ496884.1 | Yunnan_China | Southern_WildBoar | sanger |
| DQ496883.1 | Yunnan_China | Southern_WildBoar | sanger |
| DQ496882.1 | Yunnan_China | Southern_WildBoar | sanger |
| DQ496881.1 | Yunnan_China | Southern_WildBoar | sanger |
| DQ496880.1 | Yunnan_China | Southern_WildBoar | sanger |
| DQ496879.1 | Yunnan_China | Southern_WildBoar | sanger |
| DQ496878.1 | Yunnan_China | Southern_WildBoar | sanger |
| DQ496877.1 | Yunnan_China | Southern_WildBoar | sanger |
| DQ496876.1 | Yunnan_China | Southern_WildBoar | sanger |
| DQ496875.1 | Yunnan_China | Southern_WildBoar | sanger |
| DQ496874.1 | Yunnan_China | Southern_WildBoar | sanger |
| DQ496873.1 | Yunnan_China | Southern_WildBoar | sanger |
| DQ496872.1 | Yunnan_China | Southern_WildBoar | sanger |
| DQ496871.1 | Yunnan_China | Southern_WildBoar | sanger |
| DQ496870.1 | Yunnan_China | Southern_WildBoar | sanger |
| DQ496869.1 | Yunnan_China | Southern_WildBoar | sanger |
| DQ496868.1 | Yunnan_China | Southern_WildBoar | sanger |
| DQ496867.1 | Yunnan_China | Southern_WildBoar | sanger |
| DQ496866.1 | Yunnan_China | Southern_WildBoar | sanger |
| DQ496865.1 | Yunnan_China | Southern_WildBoar | sanger |
| DQ496864.1 | Yunnan_China | Southern_WildBoar | sanger |
| DQ496863.1 | Yunnan_China | Southern_WildBoar | sanger |
| DQ496862.1 | Yunnan_China | Southern_WildBoar | sanger |
| DQ496861.1 | Yunnan_China | Southern_WildBoar | sanger |
| DQ496860.1 | Yunnan_China | Southern_WildBoar | sanger |
| DQ496859.1 | Yunnan_China | Southern_WildBoar | sanger |
| DQ496858.1 | Yunnan_China | Southern_WildBoar | sanger |
| DQ496857.1 | Yunnan_China | Southern_WildBoar | sanger |
| DQ496856.1 | Yunnan_China | Southern_WildBoar | sanger |
| DQ496855.1 | Yunnan_China | Southern_WildBoar | sanger |
| DQ496854.1 | Yunnan_China | Southern_WildBoar | sanger |
| DQ496853.1 | Yunnan_China | Southern_WildBoar | sanger |
| DQ496852.1 | Yunnan_China | Southern_WildBoar | sanger |
| DQ496851.1 | Yunnan_China | Southern_WildBoar | sanger |
| DQ496850.1 | Yunnan_China | Southern_WildBoar | sanger |
| DQ496848.1 | Vietnam | Southern_WildBoar | sanger |
| DQ496847.1 | Vietnam | Southern_WildBoar | sanger |
| DQ496846.1 | Sichuan_China | Southern_WildBoar | sanger |
| DQ496845.1 | Sichuan_China | Southern_WildBoar | sanger |
| DQ496844.1 | Sichuan_China | Southern_WildBoar | sanger |
| DQ496843.1 | Sichuan_China | Southern_WildBoar | sanger |
| DQ496832.1 | Sichuan_China | Southern_WildBoar | sanger |
| DQ496831.1 | Guizhou_China | Southern_WildBoar | sanger |
| DQ496830.1 | Guizhou_China | Southern_WildBoar | sanger |
| DQ496829.1 | Guizhou_China | Southern_WildBoar | sanger |
| DQ496828.1 | Guizhou_China | Southern_WildBoar | sanger |
| DQ496827.1 | Guizhou_China | Southern_WildBoar | sanger |
| DQ496826.1 | Guizhou_China | Southern_WildBoar | sanger |
| DQ496825.1 | Laos | Southern_WildBoar | sanger |
| DQ496824.1 | Laos | Southern_WildBoar | sanger |
| DQ496823.1 | Laos | Southern_WildBoar | sanger |
| DQ496822.1 | Laos | Southern_WildBoar | sanger |
| DQ496821.1 | Laos | Southern_WildBoar | sanger |
| DQ496820.1 | Laos | Southern_WildBoar | sanger |
| DQ496819.1 | Jiangxi_China | Southern_WildBoar | sanger |
| DQ496818.1 | Jiangxi_China | Southern_WildBoar | sanger |
| DQ496817.1 | Jiangxi_China | Southern_WildBoar | sanger |
| DQ496816.1 | Jiangxi_China | Southern_WildBoar | sanger |
| DQ496815.1 | Jiangxi_China | Southern_WildBoar | sanger |
| DQ496814.1 | Hainan_China | Southern_WildBoar | sanger |
| DQ496813.1 | Hainan_China | Southern_WildBoar | sanger |
| DQ496812.1 | Hainan_China | Southern_WildBoar | sanger |
| DQ496811.1 | Hainan_China | Southern_WildBoar | sanger |
| DQ496810.1 | Hainan_China | Southern_WildBoar | sanger |
| DQ496809.1 | Hainan_China | Southern_WildBoar | sanger |
| DQ496808.1 | Hainan_China | Southern_WildBoar | sanger |
| DQ496807.1 | Hainan_China | Southern_WildBoar | sanger |
| DQ496806.1 | Hainan_China | Southern_WildBoar | sanger |
| DQ496805.1 | Hainan_China | Southern_WildBoar | sanger |
| DQ496804.1 | Hainan_China | Southern_WildBoar | sanger |
| DQ496803.1 | Hainan_China | Southern_WildBoar | sanger |
| DQ496802.1 | Hainan_China | Southern_WildBoar | sanger |
| DQ496801.1 | Hainan_China | Southern_WildBoar | sanger |
| DQ496800.1 | Hainan_China | Southern_WildBoar | sanger |
| DQ496799.1 | Hainan_China | Southern_WildBoar | sanger |
| DQ496798.1 | Hainan_China | Southern_WildBoar | sanger |
| DQ496797.1 | Hainan_China | Southern_WildBoar | sanger |
| DQ496796.1 | Hainan_China | Southern_WildBoar | sanger |
| DQ496795.1 | Hainan_China | Southern_WildBoar | sanger |
| DQ496794.1 | Hainan_China | Southern_WildBoar | sanger |
| DQ496793.1 | Hainan_China | Southern_WildBoar | sanger |
| DQ496787.1 | Fujian_China | Southern_WildBoar | sanger |
| DQ496786.1 | Fujian_China | Southern_WildBoar | sanger |
| DQ496785.1 | Fujian_China | Southern_WildBoar | sanger |
| DQ496784.1 | Fujian_China | Southern_WildBoar | sanger |
| DQ496783.1 | Fujian_China | Southern_WildBoar | sanger |
| DQ496782.1 | Fujian_China | Southern_WildBoar | sanger |
| DQ496781.1 | Fujian_China | Southern_WildBoar | sanger |
| DQ496780.1 | Fujian_China | Southern_WildBoar | sanger |
| DQ496779.1 | Fujian_China | Southern_WildBoar | sanger |
| DQ496778.1 | Fujian_China | Southern_WildBoar | sanger |
| DQ496777.1 | Fujian_China | Southern_WildBoar | sanger |
| DQ496776.1 | Fujian_China | Southern_WildBoar | sanger |
| DQ496775.1 | Fujian_China | Southern_WildBoar | sanger |
| DQ496774.1 | Fujian_China | Southern_WildBoar | sanger |
| DQ496773.1 | Fujian_China | Southern_WildBoar | sanger |
| DQ496743.1 | Yunnan_China | Southern_WildBoar | sanger |
| DQ496741.1 | Yunnan_China | Southern_WildBoar | sanger |
| FM244688.1 | Thailand | Southern_WildBoar | sanger |
| FM244687.1 | Thailand | Southern_WildBoar | sanger |
| FM244686.1 | Thailand | Southern_WildBoar | sanger |
| FM244685.1 | Thailand | Southern_WildBoar | sanger |
| FM244684.1 | Thailand | Southern_WildBoar | sanger |
| FM244683.1 | Thailand | Southern_WildBoar | sanger |
| KX982644.1 | Vietnam | Southern_WildBoar | sanger |
| KX982643.1 | Vietnam | Southern_WildBoar | sanger |
| KX982642.1 | Vietnam | Southern_WildBoar | sanger |
| KX982641.1 | Vietnam | Southern_WildBoar | sanger |
| KX982640.1 | Vietnam | Southern_WildBoar | sanger |
| KX982639.1 | Vietnam | Southern_WildBoar | sanger |
| KX982638.1 | Vietnam | Southern_WildBoar | sanger |
| KX982637.1 | Vietnam | Southern_WildBoar | sanger |
| KX982636.1 | Vietnam | Southern_WildBoar | sanger |
| EF545585.1 | Yunnan_China | Southern_WildBoar | sanger |
| EF545586.1 | Yunnan_China | Southern_WildBoar | sanger |
| EF545584.1 | Vietnam | Southern_WildBoar | sanger |
| EF545579.1 | Jiangxi_China | Southern_WildBoar | sanger |
| EF545573.1 | Yunnan_China | Southern_WildBoar | sanger |
| EF545572.1 | Hainan_China | Southern_WildBoar | sanger |
| EF545571.1 | Fujian_China | Southern_WildBoar | sanger |
| EF545570.1 | Fujian_China | Southern_WildBoar | sanger |
| EF545569.1 | Fujian_China | Southern_WildBoar | sanger |
| EF545568.1 | Yunnan_China | Southern_WildBoar | sanger |
| KP681245.1 | Hainan_China | Southern_WildBoar | sanger |
| KC505411.1 | Southwest_China | Southern_WildBoar | sanger |
| AB041466.1 | Yunnan_China | Southern_WildBoar | sanger |
| DQ779420.1 | Shanghai_China | Southern_WildBoar | sanger |
| FJ601521.1 | Zhejiang_China | Southern_WildBoar | sanger |
| DQ496303.1 | Yunnan_China | Southern_WildBoar | sanger |
| DQ496304.1 | Yunnan_China | Southern_WildBoar | sanger |
| FJ601525.1 | Jiangxi_China | Southern_WildBoar | sanger |
| AY884683.1 | Hunan_China | Southern_WildBoar | sanger |
| FJ601522.1 | Zhejiang_China | Southern_WildBoar | sanger |
| AY884610.1 | Sichuan_China | Southern_WildBoar | sanger |
| AY884685.1 | Sichuan_China | Southern_WildBoar | sanger |
| DQ496732.1 | Vietnam | Southern_WildBoar | sanger |
| DQ496733.1 | Vietnam | Southern_WildBoar | sanger |
| AY884712.1 | Myanmar | Southern_WildBoar | sanger |
| AY884695.1 | Myanmar | Southern_WildBoar | sanger |
| AY884623.1 | Myanmar | Southern_WildBoar | sanger |
| AY884647.1 | Myanmar | Southern_WildBoar | sanger |
| AY884629.1 | Myanmar | Southern_WildBoar | sanger |
| DQ779411.1 | Myanmar | Southern_WildBoar | sanger |
| DQ779399.1 | Vietnam | Southern_WildBoar | sanger |
| DQ779410.1 | Thailand | Southern_WildBoar | sanger |
| AY884640.1 | Sichuan_China | Southern_WildBoar | sanger |
| FJ601523.1 | Hainan_China | Southern_WildBoar | sanger |
| FJ601524.1 | Hainan_China | Southern_WildBoar | sanger |
| SChineseWB10 | South China | Southern_WildBoar | NGS local assembly |
| SChineseWB13 | South China | Southern_WildBoar | NGS local assembly |
| SChineseWB103 | South China | Southern_WildBoar | NGS local assembly |
| SChineseWB205 | South China | Southern_WildBoar | NGS local assembly |
| SChineseWB01 | South China | Southern_WildBoar | NGS local assembly |
| SChineseWB02 | South China | Southern_WildBoar | NGS local assembly |
| SChineseWB29U12 | South China | Southern_WildBoar | NGS local assembly |
| SChineseWB29U14 | South China | Southern_WildBoar | NGS local assembly |
| SChineseWB29U16 | South China | Southern_WildBoar | NGS local assembly |
